# Change of *d*-irection: current limitations and future directions in psychological meta-analysis

**DOI:** 10.3389/fpsyg.2026.1717798

**Published:** 2026-02-13

**Authors:** Irene Alfarone, Matthias Gondan

**Affiliations:** Department of Psychology, University of Innsbruck, Innsbruck, Austria

**Keywords:** meta-analysis, missing data, multiple imputation, multivariate, standardized effect sizes

## Abstract

Meta-analysis is a statistical tool used to combine the results of multiple studies to answer a research question. In psychology, effects are often measured on different scales (i.e., with different units), and their aggregation is not trivial. The problem is commonly solved using standardized effect sizes such as Cohen’s *d*. Despite being widely adopted, this approach is flawed. The misunderstanding is that standardized measures are dimensionless by definition—two *d* do not share the same dimension, as they do not have any. The present work explores alternative approaches to meta-analysis: Multivariate meta-analysis jointly models correlated outcomes while preserving their original unit; Imputation techniques treat the different outcome measures as a missing data problem. We evaluate these approaches through Monte Carlo simulation and an application to real data from psychotherapy studies. Results confirm that under missingness at random, multivariate meta-analysis provides meaningful and precise estimates. Imputation techniques offer an even more flexible alternative for dealing with non-ignorable missing outcome measures. The findings encourage the adoption of multivariate and imputation-based meta-analysis techniques to reduce bias, avoid research waste, and enhance the interpretability of psychological findings.

## 1 Introduction

“*[…] psychologists have to start respecting the units they work with, or develop measurement units they can respect enough so that researchers in a given field or subfield can agree to use them. In this way, there can be hope that researchers’ knowledge can be cumulative.*” (Cohen, 1994, p. 1001)

“*Being so disinterested in our variables that we do not care about their units can hardly be desirable.*” (Tukey, 1969, p. 89)

Meta-analysis (MA) is a statistical approach designed to quantitatively synthesize the results of multiple studies to answer a research question. MAs can be conducted using aggregate data (e.g., mean differences, risk ratios) or by reanalyzing raw data from the studies; the latter is referred to as individual participant data (IPD) meta-analysis. The term “meta-analysis” was first coined by Glass (1976); however, the roots of this statistical technique can be traced back to Pearson’s (1904) report on enteric fever inoculation. Since then, meta-analysis has become a widely adopted tool across disciplines, including, but not limited to, medicine, psychology, education, and economics (Ahn et al., 2012; Havránek et al., 2020; Polanin et al., 2020; Signore and Campagna, 2023).

Despite its widespread adoption, meta-analysis faces intrinsic limitations, with heterogeneity being the main challenge. Heterogeneity refers to variability in study designs, participants, interventions, outcomes, and measurement instruments (Stogiannis et al., 2023). The Cochrane Handbook for Systematic Reviews of Interventions (Higgins et al., 2024) defines three primary sources of heterogeneity: clinical heterogeneity (differences in outcomes, participants, and interventions), methodological heterogeneity (differences in study design and risk of bias), and statistical heterogeneity, which emerges as a result of the first two sources. Although statistical measures such as Cochran’s *Q* and the *I*^2^ statistic are commonly used to quantify heterogeneity, they do not fully capture the complexity of the issue (Ioannidis, 2008; Holzmeister et al., 2024). For instance, a low *I*^2^ may not necessarily imply that interventions are comparable (Hemming et al., 2021). To properly analyze heterogeneity and gain valuable insights into its patterns, several authors suggest that researchers should use a broad set of quantitative and qualitative tools for a more nuanced and tailored overview of the phenomenon of interest (Parr et al., 2019; Choi and Kang, 2025).

Among the many sources of heterogeneity, a particularly critical challenge in psychological meta-analyses lies in the proliferation of measures (Elson et al., 2023). Even fundamental constructs such as depression are assessed using many different instruments across studies, each with its own psychometric properties and theoretical backgrounds. As of September 2025, the APA PsycTests database contained a formidable number of 80,447 entries, encompassing tests, questionnaires, rating scales, and surveys (American Psychological Association, 2025a). Therefore, studies that supposedly assess the same construct may do so by adopting different instruments.

The most common approach to circumvent measurement heterogeneity is the use of standardized mean differences (SMDs), typically Cohen’s *d* (Cohen, 1969), Hedges’ *g* (Hedges, 1981), or Glass’ Δ (Glass, 1976). These dimensionless effect size metrics, calculated as the mean difference divided by some standard deviation (e.g., pooled variance for Cohen’s *d*), are widely assumed to enable direct comparison across studies that use different scales (i.e., measurement instruments). In Cooper and Hedges (1994), two main justifications are provided for this practice: (1) outcomes can simply be linear transformations of each other (e.g., US dollars and Euro in economic studies); (2) Cohen’s *d* can be interpreted as the overlap between two distributions, even if the outcomes measure related but distinct constructs.

Argument (1) is straightforward: if all studies measure the same outcome on different transformations of the same scale, they can be converted into one another. Strictly speaking, standardization is not even necessary for this conversion. On the other hand, argument (2) has received strong criticism from both statistical and applied standpoints. First, to interpret Cohen’s *d* in terms of distributional overlaps, the data must be normally distributed. This condition is often violated in psychological studies (Micceri, 1989), and its violation can lead to biased interpretations (Sun and Cheung, 2020). Second, several authors have shown that variations in study design can bias Cohen’s *d*, making it unreliable for cross-study comparisons when the groups have different variances (Hunter and Schmidt, 1990; Harrer et al., 2021). For instance, comparing two studies with identical scales and mean differences but unequal variances, leads to different Cohen’s *d* values despite the same raw effect. Such sensitivity to variance differences compromises the comparability of standardized effect sizes across studies (Morris and DeShon, 2002; Bond et al., 2003; Baguley, 2009).

The above issues can sometimes be addressed with statistical workarounds (e.g., transformations for non-normality, use of the control group’s standard deviation for standardization in Glass’ Δ). However, at the practical level, greater limitations arise and require broader considerations. Standardization rests on the tacit assumption that because effect sizes are dimensionless, they are directly comparable across studies. This assumption is fundamentally flawed. A standardized effect size is *dimensionless* by definition; having no metric does not imply having the same metric. For illustration, consider two hypothetical studies: one measures an effect in meters, the other in kilograms. Standardizing each effect size removes the original units, yielding two dimensionless numbers that cannot be meaningfully combined. The mere fact that both are unitless does not create comparability: averaging “two SDs of length,” for instance, with “three SDs of weight” is non-sensical, regardless of the statistical considerations raised above.

In psychology, we rarely face the issue of combining studies measuring effects in kilograms and meters. However, even within the measurement of the same psychological construct (i.e., depression), different instruments measure different aspects. For example, recent work by Fried et al. (2022) illustrates the various sets of symptoms targeted by the most commonly adopted depression rating scales. Aggregating these measures into one dimensionless number hides differences in meaning across scales and may lead to a loss of information and potential bias. A compelling illustration of the problem is the study by Cuijpers et al. (2010), who examined whether self-reported and clinician-rated depression instruments yield comparable outcomes in psychotherapy studies. Their findings highlight that even highly correlated measures are not equivalent in measuring treatment efficacy (in particular, clinician ratings reported greater treatment efficacy than self-reports). That self-reports differ from clinician ratings is not surprising, as they rely on different sets of symptoms, different precision, different levels of insight, and, obviously, different levels of diagnostic experience.

This paper is aimed at empirical researchers and shows how to overcome the limitations of standardized effect sizes using two statistical approaches with simulated and real data. The first involves performing a multivariate instead of a univariate meta-analysis. Multivariate meta-analysis extends the univariate framework by jointly modeling multiple outcomes, explicitly accounting for their correlation (Kalaian and Raudenbush, 1996; Riley et al., 2015). As an alternative strategy, when within-study correlations are unavailable and studies report different yet overlapping sets of outcomes, we propose to reframe meta-analysis as a missing data problem and the use of outcome imputation (Rubin, 1987) as a practical workaround. The added value of this approach is twofold: it provides a flexible way to include studies when the correlations cannot be reliably retrieved and naturally enables sensitivity analyses with delta adjustment to deal with situations in which the assumptions underlying missing data imputation are not met. The paper presents the theoretical and methodological background of these techniques, but readers primarily interested in their application can concentrate directly on the simulated and applied examples, their results, and the practical recommendations. As a motivating example, we reanalyze the dataset of Cuijpers et al. (2010) to highlight the real-world implications of measurement heterogeneity in psychotherapy research and assess the performance of these approaches through a simulation study.

The remainder of this paper is organized as follows: Section 2 describes the conceptual and statistical background of univariate and multivariate meta-analysis, presents the design and implementation of the simulation study, and discusses the reanalysis of Cuijpers et al. (2010)’s dataset. Section 3 displays the results of the simulation and case study. Section 4 discusses the findings from the simulations and the real-world case study, highlighting their implications for practice and future research. We conclude with recommendations for conducting meta-analyses in psychology when outcomes are measured on different scales.

## 2 Methods

### 2.1 Overview of meta-analytical methods

This section introduces the three meta-analytic approaches evaluated in this paper: univariate meta-analysis, multivariate meta-analysis, and outcome imputation followed by univariate analysis.

#### 2.1.1 Univariate meta-analysis

In a meta-analysis model, let *S* denote the number of studies, with s=1,…,S . For study s , ${\hat{\theta }}_{s}$ represents the observed treatment effect estimate, and ${\hat{\sigma }}_{s}^{2}$ is the estimate of $\mathrm{Var}({\hat{\theta }}_{s})$ based on the sampling variance. The fixed-effects model assumes a true effect size *θ*, with differences between studies resulting solely from (usually, normally distributed) sampling error:

$${\hat{\theta }}_{s}=\theta +\varepsilon_{s},\;\varepsilon_{s}\sim N(0,\sigma_{s}^{2}).$$

Conversely, in a random-effects model, we assume a distribution of true effect sizes, of which we typically estimate the mean (Borenstein et al., 2010). This implies that the differences between studies are due to a combination of sampling error ( $\varepsilon_{s}$ ) and genuine variation among effects ( $\zeta_{s}$ ).

$${\hat{\theta }}_{s}=\theta_{s}+\zeta_{s}+\varepsilon_{s},\;\varepsilon_{s}∼N(0,\sigma_{s}^{2}),\;\zeta_{s}∼N(0,\tau^{2})$$

$\tau^{2}$ is the between-study variance that can be estimated, for instance, with maximum likelihood, restricted maximum likelihood, or the methods of moments. For $\tau^{2}=0$ , the random-effects model reduces to the fixed-effects model. The between-study variance $\zeta_{s}$ is not an intrinsic property of study *s*; rather, if the study were repeated, $\zeta_{s}$ would be another random draw from $N(0,\tau^{2})$ . This is known as the exchangeability assumption (Schwarzer et al., 2015).

The pooled estimate of the effect of interest is usually obtained using an inverse-variance weighting approach:

$${\hat{\theta }}_{\text{pooled}}=\frac{\sum_{s}w_{s}{\hat{\theta }}_{s}}{\sum_{s}w_{s}},$$

with weights $w_{s}=1/{\hat{\sigma }}_{s}^{2}$ for the fixed-effects model and $w_{s}=1/({\hat{\sigma }}_{s}^{2}+{\hat{\tau }}^{2})$ for the random-effects model. The choice of a suitable meta-analytic model depends on the characteristics of the study, the data, and the population. For a comprehensive overview of the topic, we refer the reader to accessible treatments by Borenstein et al. (2007), as well as Rice et al. (2018) for the ongoing debate regarding the suitability of a random- or fixed-effects model for particular meta-analytic problems. To tackle certain types of heterogeneity, a random-effects model should be used. However, the converse is not true. Adopting a random-effects model is not a universal solution to all kinds of heterogeneity problems (e.g., Higgins et al., 2009).

Univariate meta-analysis can be implemented in several distinct scenarios. In a first scenario, all studies report same outcome measure. In this case, there is no practical need to transform the effect estimates, as the studies can be meta-analyzed in their original units (e.g., Tseng et al., 2023). A second scenario occurs when all studies report the same primary outcome measure, while some also report one or more secondary outcomes. When performing a univariate meta-analysis, the researcher may decide to focus solely on the primary outcome, leaving out the information from the secondary outcomes. However, in this case, the relationship between primary and secondary outcomes would be overlooked, which is problematic on its own since information is lost, but especially problematic if the results on the primary and secondary outcomes are in contradiction (Baldwin et al., 2014).

More commonly, studies report different measures for both primary and secondary outcomes. Consider the case we will present in the simulation study, where studies report the outcome of interest on two scales: a clinician rating and a self-report scale. Researchers may either (1) analyze each scale separately or (2) convert the outcomes into SMDs and then combine these SMDs in a single meta-analysis. The first option leaves parts of the data unused, resulting in separate, isolated, and possibly underpowered analyses. In contrast, the second option relies on abstract, unitless aggregate measures, which complicates interpretation and can lead to biased results (see Section 1 and the example by Cuijpers et al., 2010).

Multivariate meta-analysis addresses these limitations by borrowing strength across correlated outcomes and avoids aggregating non-comparable measures into a single standardized one. This approach is described in the following section.

#### 2.1.2 Multivariate meta-analysis

As Bland (2011, p. 2) states: “[…] many clinical studies have more than one outcome variable; this is the norm rather than the exception. These variables are seldom independent and so each must carry some information about the others. If we can use this information, we should.” Multivariate meta-analysis was proposed by Raudenbush et al. (1988), while van Houwelingen et al. (1993) provided a bivariate random specification of the model and Arends et al. (2003) extended the bivariate to a three-variate model to study the effect of surgery on stroke-free survival. The multivariate model allows for a joint estimation of multiple outcomes, accounting for the correlation among them (Kalaian and Raudenbush, 1996). Multivariate meta-analyses are widely adopted in fields like medicine, where joint modeling of outcomes is necessary because of the inherent nature of physical phenomena, as seen in the meta-analysis of diastolic and systolic blood pressure (Riley et al., 2015).

Multivariate meta-analysis can be particularly practical in psychology, as several validated instruments often exist to assess the same construct (Schwarzer et al., 2015; American Psychological Association, 2025b). Moreover, many studies evaluate multiple outcomes with the same sample (Tyler et al., 2011). Constructs measured on different scales, yet with the same participants, lead to a natural correlation between measurements in the same study that can be aggregated to provide a more precise estimate.

One of the main features of multivariate meta-analysis is its ability to accommodate unbalanced datasets in which not all studies report the same set of outcomes. In other words, the studies included in multivariate meta-analysis do not have to present all the outcomes individually, as the method can accommodate “missing” outcomes at the study level. This missingness is typically by design; that is, authors deliberately opted not to include certain outcomes initially. More generally, if missingness can be assumed to occur at random (Missing At Random, MAR), the relationships observed in studies with complete outcomes also hold in studies with incomplete sets of outcomes (Riley, 2009).

For clarity, we present here the bivariate case. Let *S* with s=1,…,S denote again the number of included studies; $\theta_{1}$ and $\theta_{2}$ are the population effects on the two outcomes; ${\hat{\theta }}_{s1}$ is the first effect estimate in study *s*; and ${\hat{\theta }}_{s2}$ is the second effect estimate. The fixed-effects model can then be represented as follows:

$${\hat{\theta }}_{s1}=\theta_{1}+\varepsilon_{s1}$$

$${\hat{\theta }}_{s2}=\theta_{2}+\varepsilon_{s2}$$

$$(\begin{array}{c} \varepsilon_{s1}\\ \varepsilon_{s2} \end{array})∼N(0,\Omega_{s}),$$

where $\;\Omega_{s}=(\begin{array}{cc} \sigma_{s1}^{2} & \sigma_{s12}\\ \sigma_{s21} & \sigma_{s2}^{2} \end{array})\;$ is the typically unknown variance–covariance matrix with $\sigma_{s12}=\sigma_{s21}$ as the within-study covariances. From this, it follows that the matrix of the weights to pool the estimates is $W_{s}={\hat{\Omega }}_{s}^{-1}$ , which is the inverse of the variance–covariance matrix.

Similar to the univariate model, in the multivariate model, the random-effects model adds other sources of variance $\zeta_{s1}$ , $\zeta_{s2}$ for both outcomes to reflect the variability between studies.

$${\hat{\theta }}_{s1}=\theta_{1}+\zeta_{s1}+\varepsilon_{s1}$$

$${\hat{\theta }}_{s2}=\theta_{2}+\zeta_{s2}+\varepsilon_{s2}$$

$$\left(\begin{array}{c} \zeta_{s1}\;\\ \zeta_{s2} \end{array})∼N(0,Z\right)$$

$$(\begin{array}{c} \varepsilon_{s1}\\ \varepsilon_{s2} \end{array})∼N(0,\Omega_{s}),$$

where $Z=(\begin{array}{cc} \tau_{1}^{2} & \tau_{12}\\ \tau_{21} & \tau_{2}^{2} \end{array})$ is the variance–covariance matrix that represents the between-study heterogeneity among outcomes. Estimation of *Z* can be carried out, for instance, via maximum likelihood, restricted maximum likelihood, or the method of moments (Schwarzer et al., 2015).

A practical challenge of the multivariate approach is that studies do not always report the within-study covariance $\Omega_{s}$ (e.g., the correlation between clinician ratings and self-reports). Kirkham et al. (2012) propose three ways to retrieve these correlations: (1) obtain the within-study correlation from individual participant data (IPD) and then use this estimate for other studies; (2) rely on experts’ opinions; (3) calculate the Pearson correlation between the raw treatment effects in studies that report both outcomes and then use the same calculated correlation for all other studies. Bayesian approaches for estimating unknown within-study correlations are also possible. For instance, Bujkiewicz et al. (2013) use external data to construct prior distributions for within-study correlations and then estimate them in a Bayesian multivariate meta-analysis. Thilan and Jayasekara (2016) propose a related “Bayesian correlation” method to model the treatment effects across studies and use a MCMC algorithm to obtain a posterior distribution that can be used directly in the meta-analysis.

Conversely, when the within-study correlations are entirely unavailable, Riley et al. (2008) and Hong et al. (2018) developed robust variance estimators that allow valid variance estimation in multivariate meta-analysis using only the marginal variances of the outcomes. These approaches rely on a sandwich-type estimator (Huber, 1967) and have been shown to provide consistent variance estimates even when the model for the within-study covariance structure is misspecified.

An alternative formulation of multivariate meta-analysis uses the framework of Structural Equation Modeling (SEM). Cheung (2008) introduced an approach that handles study-level effect sizes as observed data and incorporates the known sampling covariance matrices as definition variables, which are observed variables that can be used to fix model parameters to specific values (Mehta and Neale, 2005). In this approach, each study is treated as an observation in the SEM framework. Parameters are then estimated via full information maximum likelihood (FIML). The method is implemented in the R package metaSEM (Cheung, 2015) and allows for flexible modeling of outcome relationships and the natural accommodation of missing outcomes at the study level under the MAR assumption.

In general, the multivariate approach can yield estimates with better statistical properties by using the correlation between outcomes to borrow strength from other data (Jackson et al., 2011). Borrowing of strength (BoS) is a statistical measure that quantifies the extent to which information from correlated outcomes contributes to the precision of estimates (Riley et al., 2017). Intuitively, BoS can also be interpreted as the percentage of trials and participants that would not have been considered if performing a univariate meta-analysis alone (Riley et al., 2017). The exception to BoS occurs when the between- and within-study correlations are zero, meaning that the outcomes are independent, or when there are no missing outcomes and the within-study variances are equal, indicating that complete and equally precise information is already available (Riley et al., 2007). Under these conditions, a multivariate approach is practically equivalent to a univariate one. Finally, adopting a multivariate model can help address outcome reporting bias (Copas et al., 2014; Frosi et al., 2015), where outcomes are selectively reported based on their significance or direction. Standard multivariate meta-analysis, however, still relies on a missing at random assumption: that is, outcome missingness is assumed to be explainable by observed values. This assumption can, however, be too restrictive. More flexible scenarios can be explored by treating heterogeneous outcomes as a missing data problem, as discussed in the next section.

#### 2.1.3 Heterogeneous outcomes as a missing data problem

In psychological meta-analysis, it is common for studies to assess the outcome of interest using different measures or to report only a subset of possible outcomes. We have already seen that the meta-analytic dataset can consist of a heterogeneous yet overlapping set of outcomes, with some observed in certain studies but not in others. This structure can be viewed as an incomplete dataset in which the missing entries do not result from data loss, but from differences in study designs and choices in outcome measurement or reporting. In this scenario, imputation of missing outcomes can offer a practical workaround to multivariate modeling in handling such incomplete datasets. The rationale is to treat these meta-analyses as missing data problems, where unreported outcomes are considered missing values “by design” or as a result of selective reporting practices (see also, e.g., Saracini and Held, 2024). Although missing data techniques are wellknown in the meta-analysis literature (e.g., Carpenter et al., 2011), their novel use here is to connect these tools to the specific problem of heterogeneous and overlapping outcome measures in meta-analysis and to offer a practical template for applied researchers, including settings with non-ignorable outcome missingness.

In his foundational work, Rubin (1976) framed missing data as the result of a causal process, distinguishing three basic cases: Missing Completely At Random (MCAR), Missing At Random (MAR), and Missing Not At Random (MNAR). Modern approaches to missing data imputation build on the theory of directed acyclic graphs and extend them to missingness graphs, or *m*-graphs, which incorporate missingness indicators and proxy variables (Mohan and Pearl, 2021). To explain these mechanisms from a meta-analytical perspective, let us consider the missing data generating process of our simulation study.

**Figure 1:**
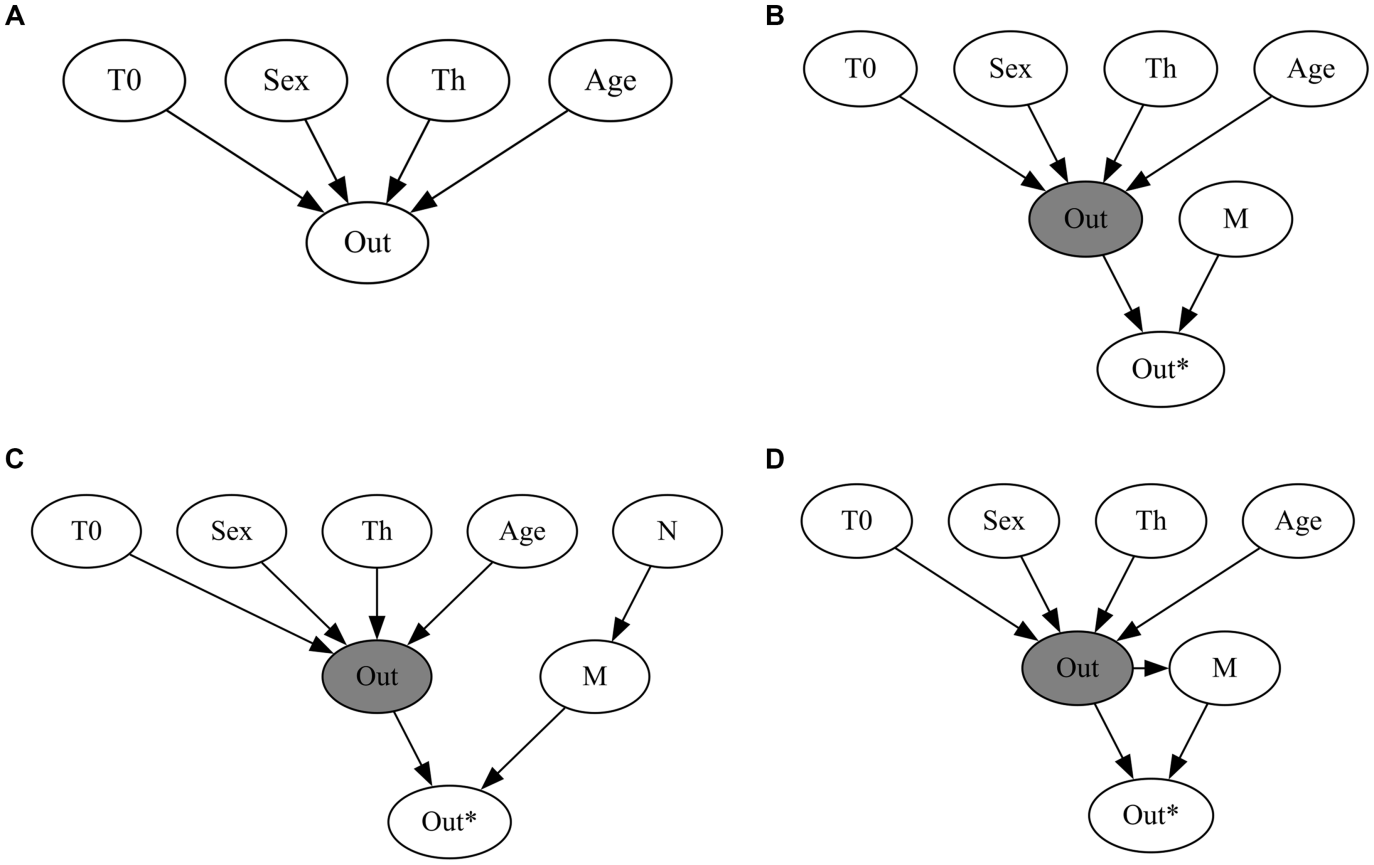
*m*-graphs displaying the missingness mechanisms adopted to generate missing data for the simulation study. T0 stands for performance at baseline; sex is the reported sex of the participants; Th stands for therapy; age is the age reported by the participants; N is the sample size; Out is the unobserved outcome variable; M is the missingness mechanism; Out* is the observed outcome variable. **(A)** Complete data. **(B)** MCAR: the missingness mechanism (M) is unrelated to any of the observed or unobserved variables. **(C)** MAR: the missingness mechanism (M) depends on the *observed* sample size (N) of the study. In smaller, therefore underfunded, studies, the CR is less likely to be reported. **(D)** MNAR: the missingness mechanism (M) depends directly on the *unobserved* values of the outcome (Out) variable.

*Missing Completely At Random*. Under MCAR, the probability of a value being missing is totally unrelated to both the unobserved and observed variables, formally:

$$P(M_{i}|Y_{i},X_{i})=P(M_{i}),$$

where *M* denotes the missingness indicator, *X* the completely observed variable, *Y* the partially observed variable, and *i* denotes the unit of analysis, in this case the study. In a multivariate meta-analysis, this implies that the missingness is pure random noise in the study collection process. A random subset of outcomes is observed without any systematic reason (Figure 1B).

*Missing At Random.* Under MAR the probability of a value being missing is conditionally independent of the unobserved variables, given the observed ones:

$$P(M_{i}|Y_{i},X_{i})=P(M_{i}|X_{i}).$$

In a meta-analysis dataset, the probability of an outcome being missing could depend on the sample size of the study. For instance, clinician rating measures would be more likely to be missing in smaller, and therefore underfunded, studies (Figure 1C).

*Missing Not At Random*. Under (focused) MNAR (Gomer and Yuan, 2021), the probability of an outcome being missing depends on the outcome itself:

$$P(M_{i}|Y_{i},X_{i})=P(M_{i}|Y_{i}).$$

In this scenario, missingness is systematically related to unobserved values—and, therefore, non-ignorable. This may occur if outcomes that do not align with the expected treatment direction or have a null effect are systematically underreported (Figure 1D).

One possible approach to address incomplete datasets is to “fill in” the missing values using information from the observed data. This strategy relies on MAR and assumes that the predictive model for the missing values is correctly specified given the observed ones. However, it has become common practice to generate several plausible values to reflect the uncertainty of the imputation process. This approach is known as Multiple Imputation (MI). MI was proposed by Rubin (1987) and is widely adopted in health research as well as in psychology (van Buuren, 2018). This method consists of three steps: (1) generate multiple complete datasets with appropriate predictive models; (2) analyze these datasets separately; (3) pool the results to obtain an overall estimate with its precise variance using Rubin’s rules (Rubin, 1987).

In the context of meta-analysis, multiple imputation can be integrated with univariate or multivariate models. For example, Viechtbauer (2022) provides an application in the R package metafor for missing covariates in univariate meta-analysis (see also Lee and Beretvas, 2023), and Carpenter et al. (2011) discuss the use of multiple imputation to assess sensitivity to selection bias in meta-analysis. Recent work has further extended multiple imputation of missing outcomes to integrate it directly with multivariate meta-analysis, as in the metavcov R package (Lu, 2023).

In addition to the analysis of MAR imputed outcomes with multiple imputation, we recommend incorporating sensitivity analysis techniques to assess the impact of potential deviations from the MAR assumption, specifically for the MNAR scenario. The approach presented here is similar to that in Fiero et al. (2017) for the imputation of MNAR data in cluster randomized controlled trials. In their work, the clusters correspond to groups randomized to the treatment arms, and the missing data occur at the outcome level within the cluster, rather than at the participant level. Similarly, in a meta-analysis with heterogeneous outcomes, the missing entries appear directly at the study level. Fiero and colleagues propose the use of pattern-mixture models (PMM) to analyze departures from MAR assumptions in longitudinal data. In PMM, the joint distribution of missing and observed data is specified through the marginal distribution of the missing data and the conditional distribution of the observed data given the missing ones:

$$f(Y_{i},M_{i})=f(Y_{i}|M_{i})f(M_{i}),$$

where $f(Y_{i},M_{i})$ means that the model’s parameters differ by missing data pattern and $f(M_{i})$ is the model that describes the pattern proportion (Little, 1993; Enders, 2022).

In practice, simple PMM implementations can be operationalized using multiple imputation with delta adjustments. Once the imputation model has been specified under MAR and the imputations have been generated, delta shift methods provide a straightforward way to introduce MNAR departures from MAR-based imputation (e.g., Leacy et al., 2017). The idea is simple: first obtain imputations under MAR, then, if there is reason to believe that the MAR imputed values are systematically biased, adjust them with a user-defined *δ* parameter, such that: ${\tilde{Y}}_{\mathrm{mis},\text{MNAR}}^{\left(m\right)}={\tilde{Y}}_{\mathrm{mis},\mathrm{MAR}}^{\left(m\right)}+\delta$ , where *m* indexes the imputed dataset and mis denotes the missing outcome entries. Afterwards, each imputed dataset is analyzed with the chosen model and the treatment effects are pooled using Rubin’s rules (Tang, 2017). Rather than trying to recover the true value under MNAR, this approach focuses on sensitivity analyses. It allows the researcher to assess how robust the conclusions are across a plausible range of MNAR assumptions, when the missingness mechanism is uncertain or non-ignorable. To present these methods, we conduct a simulation study. Below, we illustrate, in a realistic setting, how multivariate and univariate meta-analyses (with and without outcome imputation) perform when synthesizing psychological studies that report different but related outcome measures under varying mechanisms of missing outcome data.

### 2.2 Simulation study

The aim of this simulation study is to illustrate, in a controlled and plausible scenario, how univariate and multivariate meta-analyses perform in estimating the therapy effect on a psychological construct measured on two different scales: a clinician rating scale (CR) and a self-report (SR) scale. In all analyses, the outcomes of interest are the unstandardized treatment effects for CR and SR. To perform such analyses, the meta-analytical dataset should have the following structure: Each row corresponds to one study and contains the unstandardized treatment effect on the CR and its standard error, the unstandardized treatment effect on the SR and its standard error, and the within-study correlation between the CR and SR treatment effects. When the treatment effect for one outcome is not available, the corresponding cell is left empty. For illustration, Table 1 shows a possible configuration of a meta-analytical dataset for multivariate meta-analysis with unstandardized outcome measures, contrasted with the dataset for a univariate meta-analysis with standardized mean differences (Table 2).

**Table 1:** Example of a meta-analytical dataset for multivariate meta-analysis that uses raw mean differences as the outcome of interest.

Study	Clinician rating		Self-report		Correlation
	MD	SE	MD	SE	
Study 1	1.52	1.77	6.61	2.30	0.64
Study 2			3.93	2.28	
Study 3	2.88	1.37			
Study 4	3.73	1.50	6.51	2.68	0.44
Study 5			4.95	2.39	

Data are obtained with the same generating process of the simulation study. MD stands for mean difference and SE stands for standard error.

**Table 2:** Example of a meta-analytical dataset for meta-analysis that uses standardized mean differences as the outcome of interest.

Study	SMD	SE	Source
Study 1	0.05	0.23	CR
Study 2	0.38	0.22	SR
Study 3	0.51	0.16	CR
Study 4	0.78	0.22	CR
Study 5	0.72	0.29	SR

Data in this table are obtained using the same generating process of the simulation study, as in Table 1. SMD is the standardized mean difference calculated as Cohen’s *d* with their respective standard error (SE). In this case, each study contains just one treatment estimate, which is expressed in a dimensionless metric rather than on the original scales. Additionally, the correlation between clinician rating (CR) and self-report (SR) is ignored.

To represent plausible missingness scenarios, we conduct simulations under four conditions: complete data, missing outcomes completely at random (MCAR), systematically missing outcomes at random (MAR; only for the CR, as smaller underfunded studies are more likely not to report a CR), and missing outcomes not at random (MNAR; negative or null results are less likely to be reported), each evaluated at 40% of total missing outcome data at the summary level. In particular, under MNAR, we generated missing outcomes using a logistic model in which the probability of missingness for CR and SR depended on the study’s estimated treatment effect, so that unfavorable effects were more likely to be unreported.

We simulate data from 50 randomized controlled trials, with sample sizes varying from 40 to 100 participants. A latent illness construct is measured at baseline using both CR and SR as indicators. Covariates such as age and sex influence the latent illness. Participants are randomly assigned to a treatment or control group. Post-treatment outcomes (CR and SR) are generated as a function of the latent illness, a study-specific treatment effect $\theta_{\mathrm{CR}}=3$ , $\theta_{\mathrm{SR}}=5$ and outcome-specific noise (higher in SR than in CR) (see Figure 1A).

For simplicity, the latent illness and residual terms are generated from normal distributions. This design choice is motivated by the desire to isolate performance differences between methods that are due to the choice of the meta-analytical model, rather than to violations of distributional assumptions. As mentioned above, standardized mean differences are sensitive to non-normality; if we distorted the outcome distributions, we would risk amplifying the statistical weaknesses of SMDs rather than highlighting the qualitative implications of using them.

To calculate the unstandardized treatment effects and the within-study correlations, following Riley et al. (2021), we fit separate ANCOVA models per study using Seemingly Unrelated Regressions (SUR; Zellner, 1962; Henningsen and Hamann, 2008). Summary-level missing data (estimates, standard errors, and correlations) were generated using the mechanisms described in the previous section and illustrated in Figure 1. Within-study correlations between the CR and SR after covariate adjustment in the multivariate analysis are obtained directly from the SUR residual correlation matrix; the estimated within-study correlations are approximately $\rho_{\mathrm{CR},\mathrm{SR}}=0.6$ . For a detailed explanation of the correlation structure between residuals in SUR, we refer the reader to Henningsen and Hamann (2008). In Supplementary material, we also provide an example in which the correlation is arbitrarily misspecified to compare the findings.

Meta-analyses on complete and incomplete datasets are conducted using the above-mentioned approaches: (A) Univariate random-effects meta-analysis performed on non-standardized outcomes with the R package metafor (Viechtbauer, 2010), (B) Multivariate random-effects meta-analysis performed with the R package mixmeta (Sera et al., 2019), (C) Multiple imputation of missing outcomes with sensitivity analysis and delta adjustments δ∈{−3,−2,−1,0} , followed by univariate meta-analysis. Outcomes are imputed using predictive mean matching from m=20 imputed dataset with the R package mice (van Buuren and Groothuis-Oudshoorn, 2011).

We performed a Monte Carlo simulation, replicating each scenario 1,000 times to obtain reliable estimates and standard errors. The missingness rate was approximately 40% per outcome, meaning that in each simulated meta-analytical dataset, 40% of CR were missing and 40% of SR were missing. Analyses were performed with R Statistical Software, version 4.5.1 (R Core Team, 2025). The complete code is available in Supplementary material. Supplementary material also contains an extended version of this simulation that explores more moderate missingness (20%) and the implications for meta-analyses with a smaller number of studies (*S* = 25). The code is written so that key parameters (e.g., number of studies, true treatment effects, missingness rates, correlation) can be easily modified, allowing applied researchers to tailor the simulation to their own settings, assess how the methods behave under conditions that mirror their data, examine the effect of misspecifying the correlations, and explore how the results could change under different missingness assumptions.

### 2.3 Case study

Cuijpers et al. (2010) conducted a meta-analysis to assess whether clinician ratings and self-reports of depression differ in estimating the efficacy of psychotherapy. They collected 48 studies with 70 different psychotherapy conditions compared to a control group. For this analysis, we considered a subset of 37 studies presented in Appendix A of the original study, in which outcomes were reported on the Hamilton Rating Scale for Depression (HRSD-17, Hamilton, 1960) and the Beck Depression Inventory (BDI, Beck et al., 1961), and the therapy group was compared to just one control. Data for the treatment group were retrieved from Cuijpers et al. (2010); data for the control group were retrieved from the METAPSY database available at: https://www.metapsy.org/database/depression-psychotherapy. When not reported there, data were drawn directly from the original papers. We were unable to identify post-treatment data for three studies. Similar to Cuijpers et al., since the studies presented multiple comparisons with the same control group, we report here the analysis with studies that, when presenting multiple comparisons, report the highest mean difference.

This case study is used as a worked example to illustrate how the multivariate and multiple imputation methods can be implemented in practice with real data. We perform univariate, multivariate, multivariate SEM (to show equivalence between the two multivariate approaches), and univariate with outcome imputation meta-analyses as described in the sections above. In line with the literature, within-study correlation was set at ρ=0.6 (Richter et al., 1998; Hajduska-Dér et al., 2022). The univariate and multivariate models were estimated via restricted maximum likelihood; under SEM, full information maximum likelihood was used as the estimation method. The outcomes of interest are the unstandardized treatment effects on the HRSD-17 and the BDI. The analyses were performed with the R packages metafor (Viechtbauer, 2010), mixmeta (Sera et al., 2019), metaSEM (Cheung, 2015), and mice (van Buuren and Groothuis-Oudshoorn, 2011). The number of imputed datasets was set to m=50 ; the imputation method was again predictive mean matching. To explore moderate deviations from MAR, we further performed MI with *δ*-adjustments ranging from δ=−1 to δ=+1. The dataset and the complete analysis code are provided in Supplementary material to facilitate replication and adaptation in applied work.

## 3 Results

This section presents the results of the simulations for the multivariate meta-analysis and the meta-analysis with imputation of missing outcomes. However, we make it clear that these simulations are not intended as substitutes for a full theoretical analysis (Morey and Davis-Stober, 2025, p. 12), yet as an example that illustrates what applied researchers can expect realistically when using the presented methods.

### 3.1 Multivariate meta-analysis

**Figure 2:**
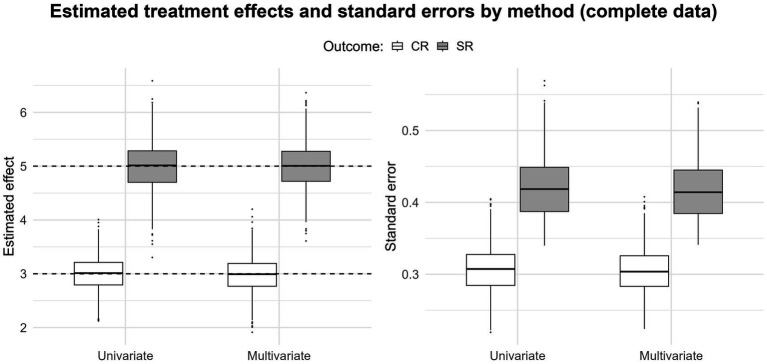
Boxplots displaying the pooled estimates and their respective standard errors for the clinician rating and self-report outcomes (complete data); the black dashed lines indicate the true value for both estimates.

The simulation study produced results consistent with pre-existing literature on multivariate meta-analysis (e.g., Riley, 2009; Jackson et al., 2011; Riley et al., 2015). Figure 2 displays the scenario for complete data. Estimates and standard errors obtained from a multivariate and univariate approach are nearly identical; the gain in precision of a multivariate approach over a univariate is negligible when there are no missing data. In practice, this means that if all outcomes are observed, moving from a univariate to a multivariate model will not practically change the results (i.e., BoS is 0%), which is in line with the findings of other studies (see also Riley et al., 2007).

**Figure 3:**
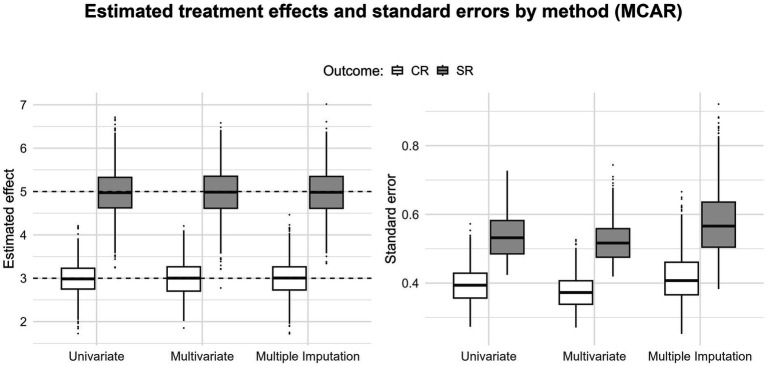
Boxplots displaying the pooled estimates and standard errors for the clinician rating and self-report outcomes (40% missing data for MCAR); the black dashed lines indicate the true value for both estimates.

**Figure 4:**
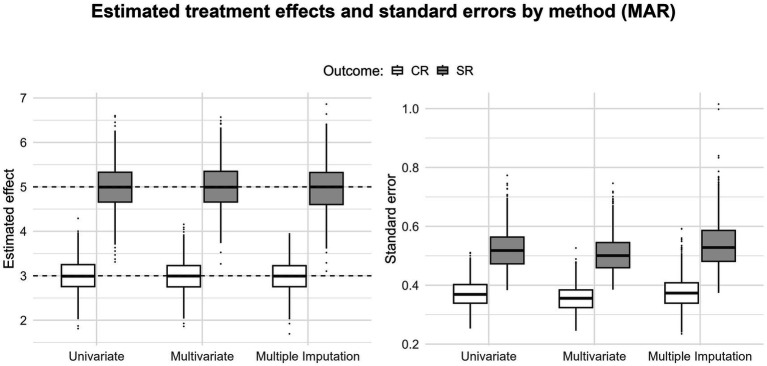
Boxplots displaying the pooled estimates and standard errors for the clinician rating and self-report outcomes (40% missing data for MAR); the black dashed lines indicate the true value for both estimates.

**Figure 5:**
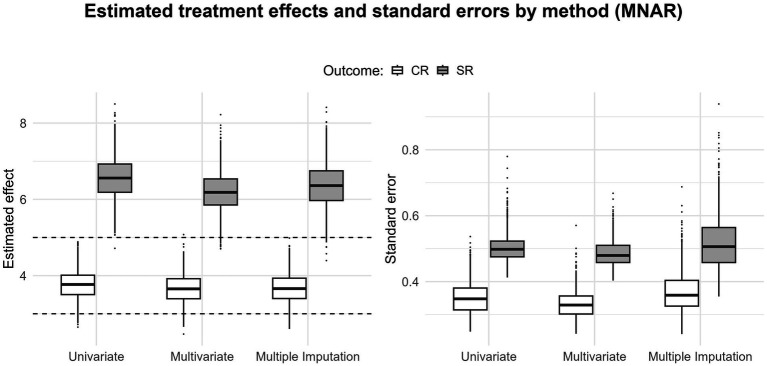
Boxplots displaying the pooled estimates and standard errors for the clinician rating and self-report outcomes (40% missing data for MNAR); the black dashed lines indicate the true value for both estimates.

When outcome measures are missing, the multivariate approach, by borrowing information from the available outcomes, leads to more precise estimates than those obtained with a univariate approach (Figures 3–5; the black dashed lines display the simulated true values). Under MCAR and MAR, the three methods yield unbiased estimates. Expectedly, under MNAR, we see upward biases for both estimates (Figure 5); this is consistent with the outcome-dependent missingness mechanism of the simulation (Figure 1D). Under MCAR and MAR, the multivariate approach gains the most in precision, as the standard errors tend to be lower in the multivariate scenario. This could reflect the situation in many applied meta-analyses where some studies report only CR or only SR. In such cases, when within-study correlations are available, a multivariate approach is a straightforward way to obtain more precise estimates (e.g., Riley et al., 2007).

### 3.2 Meta-analysis by multiple imputation

The second alternative for dealing with heterogeneous outcome measures in meta-analysis is to treat it as a missing data problem. Figure 4 shows that imputing MAR outcomes on the CR with multiple imputation leads to estimates and standard errors similar to those obtained with multivariate meta-analysis. From an applied perspective, this suggests that when studies do not report the outcome of interest measured on a common scale, the missing outcomes can be imputed under a MAR assumption, allowing the meta-analysis to be carried out on the completed dataset. Of course, when the missingness mechanism is MNAR, both the imputed values and the resulting estimates are biased, which motivates the use of sensitivity analyses based on delta adjustments.

**Figure 6:**
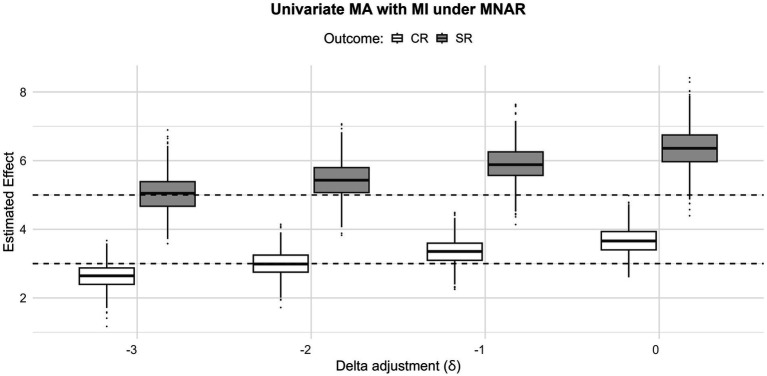
Sensitivity analysis for the MNAR imputed outcomes with delta adjustments δ∈{−3;−2;−1;0}, the black dashed lines indicate the true value for both estimates.

Figure 6 shows the delta adjustments applied to MAR-imputed outcomes analyzed with univariate meta-analysis. In this example, we vary the delta parameter to explore how treatment effect estimates change when the MAR-imputed values are systematically shifted. The goal of this adjustment is to approximate a “jump-to-reference” strategy (Carpenter et al., 2013), where the reference is the effect of the control group for the suspected biased estimates. Figure 6 indicates that when shifting the MAR toward the null hypothesis, the delta adjustment allows for a proper recovery of the true value. In general, such an approach allows the user to reveal how sensitive the results are to departures from MAR and assess plausible bounds of bias across different values to reflect different assumptions. The full coverage probabilities and biases for all methods and missingness mechanisms are reported in Appendix A.

### 3.3 Case study

The re-analysis of Cuijpers et al.’s dataset shows an interesting application of the above-mentioned techniques to a real case study. The multivariate meta-analyses conducted with mixmeta (Sera et al., 2019) and metaSEM (Cheung, 2015) report consistent estimates. In both analyses, the standard errors for the BDI are marginally lower in the multivariate meta-analysis compared to the univariate meta-analysis. This shows some moderate borrowing of strength across outcomes, although the number of missing outcomes is not too high (19% missing BDI, 14% missing HRSD). The results of the univariate meta-analyses with outcome imputation report slightly different, yet consistent results with those produced by the multivariate meta-analyses (see Table 3). However, for sensitivity analysis, we suggest using different imputation methods to prove consistency of results across several conditions.

**Table 3:** Comparison of meta-analyses of psychotherapy studies (standard errors are displayed in the parenthesis).

Outcome	Univariate	Multivariate	Multivariate SEM	Univariate imputation
BDI	−7.23 (0.95)	−6.73 (0.91)	−6.73 (0.91)	−6.94 (0.88)
HRSD-17	−6.36 (0.62)	−6.34 (0.61)	−6.34 (0.60)	−6.28 (0.61)

Furthermore, to explore moderate departures from the Missing At Random (MAR) assumption, we performed multiple imputation with delta adjustments ranging from −1 to +1. The pooled BDI effect size varied from −7.12 (SE = 0.87) at *δ* = −1 to −6.74 (SE = 0.89) at *δ* = +1. For the HRSD outcome, pooled estimates ranged from −6.41 (SE = 0.61) to −6.15 (SE = 0.62). These results suggest that the main conclusions are robust to moderate violations of the MAR assumption. It is important to note that the missing outcomes in Cuijpers et al.’s dataset are not a product of an MNAR mechanism such as selective reporting, but a simple MCAR or MAR. In Cuijpers et al.’s study, the unreported outcomes are those from studies that adopted a different version of the BDI or the HRSD-17. In this setting we therefore focused on small shifts as the missingness could reasonably be assumed by design, and the delta adjustment of the multiply imputed values simply informs the robustness of the pooled results.

## 4 Discussion

The present study shows two main alternatives to the use of SMDs in meta-analysis when outcomes are reported on different scales. Standardized mean differences, such as Cohen’s *d*, are adopted under the assumption that standardization makes outcomes comparable. However, as Cohen (1994), Tukey (1969), and subsequent scholars have noted, this assumption is intrinsically flawed. The dimensionlessness of SMDs does not make effect sizes comparable. As a matter of fact, pooling effect sizes that have different units (e.g., different symptoms, purposes, theoretical backgrounds) undermines the precision of the pooled estimate and the overall interpretability of the meta-analytical result.

The first alternative strategy to SMDs we presented is multivariate meta-analysis. In existing research, multivariate meta-analysis has been used for joint modeling of primary and secondary outcomes in psychotherapy studies (e.g., anxiety and depression) and correlated outcome measures such as arithmetic and literature skills (e.g., Raudenbush et al., 1988; Frosi et al., 2015; Daros et al., 2021; Savatsomboon et al., 2024). In this study, we promote its extension to address issues of measurement heterogeneity, that is, when the same phenomenon of interest is measured with different instruments.

To do so, we showed their implementation via a simulation study and a re-analysis of a published meta-analytical dataset. The simulation yields results consistent with those of previous studies on multivariate vs. univariate analyses (Riley et al., 2007, 2017; Riley, 2009) and illustrates how these methods perform in plausible meta-analytic scenarios that mirror applied psychological research. In particular, univariate and multivariate meta-analysis models produce nearly identical results when all outcome data are complete. Under both MCAR and MAR scenarios, outcomes were missing in selected studies, and multivariate meta-analysis yielded more precise estimates by borrowing strength from the observed outcomes. When outcomes were MNAR, bias emerged in both CR and SR estimates: CR and SR estimates were biased upwards, reflecting the missingness mechanism. Furthermore, multivariate meta-analysis and multiple imputation of missing outcomes under MNAR produced less biased estimates than those from a univariate analysis.

As a second alternative to SMDs, we frame meta-analysis as a missing data problem. For this purpose, we used multiple imputation with predictive mean matching, as implemented in the mice R package, to impute the missing outcome values under the three different simulated missing data mechanisms. Importantly, multiple imputation under MAR produced results comparable to those of the multivariate approach, supporting its use as a practical alternative to explore the robustness of results, especially when within-study correlations are unknown and difficult to estimate with other methods (see, for instance, Riley’s robust variance estimator; Riley et al., 2008; Hong et al., 2018). In this sense, the main value of the imputation approach is not only to reproduce the results of the multivariate analysis but also to provide an accessible alternative that does not require imputing correlations. Building on Fiero et al. (2017), we further proposed using delta adjustments to perform sensitivity analyses to explore deviations from the MAR assumption. The results from the simulation show that choosing the correct set of delta values allows for estimates closer to the true value (see Figure 6) and, more generally, that varying delta makes explicit how robust the pooled conclusions are to plausible departures from MAR.

A key advantage of pattern-mixture models is that they make the assumptions about the missing data completely explicit, unlike selection models where model specification is a non-trivial issue (Enders, 2022). Naturally, determining an appropriate delta shift is challenging because it involves unmeasured outcomes and therefore cannot be retrieved from observed data alone. For this purpose, White et al. (2007) discuss expert elicitation as a crucial step to define plausible adjustments and to motivate the range of *δ* values used in the sensitivity analysis. Alternatively, researchers can perform a tipping point analysis in which *δ* is gradually increased (or decreased) until the substantive conclusion changes, and then evaluate whether the required shift is plausible in the scenario considered (Gorst-Rasmussen and Tarp-Johansen, 2022). In this sense, performing the analyses under varying deltas provides the additional benefit of exploring and testing a full range of potential reasons for missing data in the meta-analysis, from moderate to extreme deviations from MAR, rather than relying on a single unverifiable adjustment.

Notably, we integrated the use of *m*-graphs (Mohan and Pearl, 2021) to formalize the assumptions about missingness and decide whether consistent estimation is possible under the assumed mechanism. By building the *m*-graph, the researcher makes explicit the assumptions regarding the missing data. If, for instance, the researcher concludes that the MAR assumption is tenable, then univariate meta-analysis followed by multiple imputation of missing outcomes or, directly, multivariate meta-analysis is possible. However, if there is insufficient evidence to justify a MAR assumption, the researcher can explore deviations from it by combining multiple imputation with delta adjustment sensitivity analyses. Such a procedure promotes transparency, strengthens the practice of using causal reasoning in research, and supports the choice of the most appropriate meta-analytical strategy.

More importantly, besides its statistical properties, multivariate meta-analysis also offers a more solid methodological framework. It allows researchers to retain the estimates’ original unit of measure, uses available data without discarding information, and provide more interpretable and less biased estimates. One of the main critiques of meta-analysis is, in fact, related to the “apples and oranges problem”, which involves combining results from studies with different features (Sharpe, 1997). Multivariate meta-analysis helps to circumvent at least part of this issue: researchers can model different measures jointly without worrying about losing the unit of measure (e.g., different symptoms for depression research) or conducting underpowered separate meta-analyses.

In this sense, the case study clearly displays the practical and theoretical tension that exists between different measures of the same construct. As shown by Fried et al. (2022), the Hamilton Rating Scale for Depression (Hamilton, 1960) and the Beck Depression Inventory (Beck et al., 1961) measure different sets of symptoms, and their sensitivity to treatment success differs (Cuijpers et al., 2010). Treating the BDI and the HRSD-17 as interchangeable using SMDs ignores their differences and can distort the interpretation of pooled estimates. By contrast, multivariate meta-analysis respects the structural heterogeneity of these outcomes while simultaneously borrowing strength from their correlation to improve precision.

This is particularly relevant for the interpretation of meta-analytical results. One reason researchers rely on SMDs is that they provide general benchmarks (small, medium, large) for interpreting intervention effects. However, if the effects are kept in their original units, the interpretation is guided by the scale itself. For the HRSD-17, for instance, a 4–6 point change is considered clinically meaningful, while a change from 7 to 12 is considered clinically substantial (Rush et al., 2021). By contrast, for the BDI-II, differences of 3 points are already considered clinically relevant, while this is naturally context-dependent (Button et al., 2015). In this sense, the pooled HRSD and BDI estimates from the case study can be directly interpreted as clinically meaningful. More generally, these alternatives to SMDs complement the recent framework on Minimal Clinically Important Differences, which aims to identify the smallest change that is likely to matter in practice (McGlothlin and Lewis, 2014; Soltaninejad et al., 2025). Reporting pooled effects in the original units can therefore make it easier for applied researchers to assess practical relevance at a glance, particularly for patient-reported outcomes (Sedaghat, 2019). Of course, as King (1986, p. 671) notes, if the scales are meaningless to begin with, interpretation is also challenging; however, standardization does not add information or meaning and therefore cannot solve the problem. For this reason, as Cohen (1994) and Tukey (1969) wrote, agreement on and interest in the units of measurement is fundamental for cumulative knowledge in psychology.

Another strategy worth discussing, as it is often adopted in meta-analysis, is to include the outcome type as a moderator. This option, in its simplicity, is appealing because it is straightforward to implement and does not require knowledge of the within-study correlations. However, in the presence of outcomes measured on different scales, it still has limitations. First, using the type of outcome as a moderator is appropriate only when effect sizes are already expressed on a common metric. If researchers want to retain the original outcome measures, as in the case study with HRSD and BDI, a univariate model with a moderator would reintroduce standardization, thereby not resolving the interpretative issues of SMDs. Second, as noted in Baek and Luo (2023), when multiple effects from the same study are included, dependence must be handled explicitly to avoid inaccurate inferences, for instance, using a multilevel model. A useful application of the moderator approach is therefore when outcomes share the same metric. For instance, when synthesizing multiple subscales from the same instrument, the moderator can inform about the variability across the different subscales or domains (e.g., physical versus psychological health-related quality of life). In other situations, a multivariate approach would still be preferable, as it preserves the unit of measure and correctly models dependencies between effects. When correlations are unavailable, or researchers want to examine departures from MAR, MI with or without sensitivity analysis can be adopted instead.

To summarize, for the applied researcher, our findings carry important implications. First, when studies assess the same construct using different instruments, such as clinician ratings and self-reports in psychotherapy research, and within-study correlations are either reported, estimable, or can be approximated from external data, multivariate meta-analysis seems to be the most appropriate methodological and analytical strategy. In these scenarios, multivariate models allow for the joint modeling of related outcomes while preserving the metric of each individual measure. This approach results in pooled estimates that are not only statistically more precise but also more interpretable for clinical and applied decision-making (e.g., Baguley, 2009). Furthermore, this analytical strategy avoids research waste, allows for the inclusion of studies with only one of the outcomes, and provides a solid strategy for addressing outcome reporting bias (as extensively shown by Copas et al., 2014; Frosi et al., 2015).

Second, we have demonstrated that multiple imputation under the MAR assumption, followed by univariate meta-analysis, can serve as a practical and flexible alternative when within-study correlations are unavailable. In such contexts, multiple imputation enables the inclusion of all available studies by imputing the missing outcomes based on the observed data structure. Furthermore, with delta adjustment sensitivity analyses, researchers can further explore the robustness of their conclusions concerning violations of the MAR assumption. This way, rather than making unverifiable assumptions about the missing data mechanism, delta adjustments offer a transparent tool to assess how results would change under plausible MNAR scenarios.

### 4.1 Limitations and future research

The experienced meta-analyst knows that encountering multiple instruments designed to measure the same construct is the rule, rather than the exception. This issue has been extensively discussed by Elson et al. (2023) who have proposed SOBER guidelines (Standardisation Of BEhavior Research) to address the proliferation and heterogeneity of psychological measurement tools.

The careful reader will have noticed that the scenario presented in this study offers a simplified representation of the complex reality of conducting meta-analyses in psychology. In our work, we focused on a bivariate meta-analytical framework to compare outcomes measured on two different scales. However, real-world applications are often far more nuanced. Extending multivariate meta-analysis beyond two outcomes is conceptually straightforward (see, e.g., Arends et al., 2003) but can be practically challenging. When datasets are too sparse (i.e., when too many different outcome measures are used and there is insufficient overlap across studies), multivariate models may fail to converge or yield unstable estimates due to a lack of information to identify the full covariance structure. In this case, rather than relying on standardized measures that may be difficult and possibly biased in interpretation, a pragmatic strategy would be to conduct separate univariate meta-analyses and explicitly acknowledge their resulting limitations (e.g., limited power, no borrowing of strength across outcomes). Alternatively, if possible, meta-analysts could also consider reducing the number of outcomes to focus on a smaller set of conceptually similar ones before fitting multivariate analyses or performing multiple imputation with missing outcomes.

A second practical challenge may arise when within-study correlations are not available. If individual participant data are not accessible, multivariate meta-analysis can still be performed, but retrieving plausible information on the correlation is non-trivial. In such scenarios, practical alternatives include expert elicitation, using correlations from related studies, or specifying Bayesian priors, as discussed above. The extent to which the chosen within-study correlations influence the analysis results can be limited (Ishak et al., 2008) and may only marginally impact the obtained estimates (see the additional analyses in Supplementary material), but their impact may also depend on several factors, such as the between-study covariance structure or the missingness rate (Riley et al., 2017). Therefore, if correlations are not available, their implications should be examined using sensitivity analyses across a range of plausible values, and, where possible, by comparing with alternative models that do not require them (Riley, 2009; Jackson et al., 2011). This recommendation also follows recent developments in multiverse meta-analysis, which encourage assessing how robust the meta-analytical conclusions are to a variety of methodological choices (Voracek et al., 2019).

Taken together, these practical challenges highlight broader methodological dilemmas in meta-analysis within psychology. When outcomes are assessed using scales that capture different facets of the same construct, should this variability be regarded merely as noise that a random-effects model can accommodate, or does it reflect deeper differences that cannot be adequately addressed by modeling statistical heterogeneity alone (for instance, with meta-regressions)? Practitioners and researchers should make careful decisions in determining which studies are sufficiently similar to be meta-analyzed. Asserting that two studies measure the same construct may not be enough to pool them together, especially when the scales refer to different aspects of the same construct. A number of authors, therefore, advocate against SMDs (e.g., Greenland et al., 1991; King, 1986; Baguley, 2009; Tukey, 1969; Jones and Waller, 2013) and in favor of multivariate MAs (e.g., Raudenbush et al., 1988; Bland, 2011; Jackson et al., 2011; Frosi et al., 2015; Riley et al., 2017).

The simulation design was intended to be informative and relatively comprehensive. However, it necessarily relied on assumptions about the data-generating process, the structure of effect sizes, and the extent and type of missingness. These assumptions may not generalize across all contexts. In particular, because the assumption of normality is often unsatisfied in psychological studies, an important direction for future research is to explore how departures from normality may influence the performance of multivariate analyses compared to meta-analyses based on standardized effect sizes. Similarly, while delta adjustment procedures can be used to explore how conclusions might vary under different plausible conditions, their utility depends on a thorough understanding of the investigated phenomenon. In the simulation scenario, we relied on the tacit assumption that we knew the true value. In real applications, however, this information is typically unavailable. Deviations from MAR can be explored, with the caveat that it is not merely a statistical problem. The expert’s opinion is the most relevant aspect in this scenario, and the objectivity of decisions is a non-trivial issue (White et al., 2007).

From a methodological point of view, further work is needed to extend the present approach beyond clinical psychology. Other fields, such as organizational psychology or environmental psychology, may present even greater heterogeneity in measurement techniques. Field-specific research could help document the prevalence and structure of measurement heterogeneity and assess the practical feasibility of applying multivariate meta-analytic strategies across disciplines.

Future studies will aim to extend the research on MAR deviations in multivariate meta-analysis to investigate whether addressing MNAR problems in a multivariate setting can provide additional insight into the precision of the estimates. Similarly, even though IPD meta-analyses are still rare in psychology, we plan to explore these aspects using IPD to provide more careful and detailed modeling of MNAR outcomes with sensitivity analysis techniques such as reference-based multiple imputation (Carpenter et al., 2013) or selection models (Heckman, 1976; Muñoz et al., 2024).

### 4.2 Conclusion

This study critically presented the limitations of using SMDs in psychological meta-analyses when outcomes are reported on different measurement scales and provided two alternatives: multivariate meta-analysis and multiple imputation of missing outcomes. Although multivariate techniques (e.g., Raudenbush et al., 1988; Jackson et al., 2011; Frosi et al., 2015; Savatsomboon et al., 2024) and missing data imputation techniques (e.g., Carpenter et al., 2011; Lu, 2023; Saracini and Held, 2024) are now well established in the meta-analytical literature, they have not, to the best of our knowledge, been used in psychology to address the heterogeneity of outcome measures across studies. Using a simulation study and a reanalysis of psychotherapy data from Cuijpers et al. (2010), we showed that multivariate models and multiple imputation under MAR can yield precise and readily interpretable estimates. Multiple imputation also allows for performing meta-analysis when correlations are not available and supports sensitivity analyses for non-ignorable missing outcomes. These methods offer robust results under various missing data scenarios and preserve the unit of measure for each outcome, better aligning with psychological theory and providing readily interpretable estimates for practitioners.

Performing meta-analysis in psychology is a non-trivial procedure. Decisions about pooling outcomes require careful theoretical justification, particularly when measurement heterogeneity reflects conceptual rather than purely statistical differences. Our results suggest that multivariate models are preferable whenever within-study correlations are available or estimable, as they preserve the unit of measure for the outcome, reduce research waste, and help address outcome reporting bias. When such correlations are not accessible, multiple imputation with sensitivity analysis represents a viable and flexible alternative.

## Data Availability

The original contributions presented in the study are included in the article/Supplementary material, further inquiries can be directed to the corresponding author.

## Appendix A

**Table A1:** Bias and coverage for all simulation settings are presented in the table below.

Method	Mechanism	CR coverage	SR coverage	CR bias	SR bias
Univariate	Complete	0.954	0.944	0.002	0.003
Univariate	MCAR	0.942	0.939	−0.006	−0.017
Univariate	MAR	0.946	0.950	−0.002	−0.013
Univariate	MNAR	0.425	0.136	0.768	1.566
Multivariate	Complete	0.941	0.944	0.019	0.037
Multivariate	MCAR	0.939	0.933	−0.002	−0.024
Multivariate	MAR	0.947	0.953	−0.014	−0.003
Multivariate	MNAR	0.488	0.298	0.667	1.207
Multiple imputation	MCAR	0.941	0.951	0.002	−0.021
Multiple imputation	MAR	0.964	0.948	−0.009	−0.024
Multiple imputation	MNAR	0.560	0.263	0.668	1.360

Method indicates the meta-analytical strategy used; Mechanism indicates the missing data mechanism used to generate the missing values; Coverage (respectively for the clinician rating, CR and self-report SR) is defined as the proportion of replicates in which the 95% confidence interval contained the true effect; Bias is calculated as the average difference between the estimated and the true effects.
