# Supplementary material for: Change of d-irection: current limitations and future directions in psychological meta-analysis
Source: Front Psychol. 2026 Feb 13;17:1717798. doi: 10.3389/fpsyg.2026.1717798 (PMC12946090; doi:10.3389/fpsyg.2026.1717798)
Supplement: Supplementary file 1 [file Data_Sheet_1.PDF]

R code for case study

2025-10-02

```
library(readxl)
library(dplyr)
d = read_xlsx("casestudy-cujipersetal-data.xlsx")
head(d)
```

```
## # A tibble: 6 x 14
##      ID Study               Psychotherapy    Nt    Nc     N `T-BDI-M-Post` `T-BDI-SD-Post`
##   <dbl> <chr>               <chr>         <dbl> <dbl> <dbl> <chr>          <chr>
## 1     1 Arean et al. (1993~ PST              28    21    49 15.7           6.9
## 2     1 Arean et al .(1993~ Reminiscence     27    21    48 16.9           9.5
## 3     2 Ayen and Hautzinge~ CBT              11    10    21 8.5            4.0
## 4     2 Ayen and Hautzinge~ Supportive       20    10    30 14.6           4.5
## 5     3 Bowers et al. (199~ CBT               8     8    16 9.0            6.1
## 6     4 Bowman, Scogin, an~ CBT              10    10    20 12.4           11.4
## # i 6 more variables: `T-HRSD-M-Post` <chr>, `T-HRSD-SD-Post` <chr>,
## #   `C-BDI-M-Post` <chr>, `C-BDI-SD-Post` <chr>, `C-HRSD-M-Post` <chr>,
## #   `C-HRSD-SD-Post` <chr>
```

```
d <- d %>%
  mutate(
    across(c(-Study, -Psychotherapy), as.numeric),
    MissHRSD=ifelse(is.na(`T-HRSD-M-Post`), "0", "1"),
    MissBDI=ifelse(is.na(`T-BDI-M-Post`), "0", "1"),
    across(c(Psychotherapy), as.factor)
  )
head(d)
```

```
## # A tibble: 6 x 16
##      ID Study               Psychotherapy    Nt    Nc     N `T-BDI-M-Post` `T-BDI-SD-Post`
##   <dbl> <chr>               <fct>         <dbl> <dbl> <dbl>          <dbl>           <dbl>
## 1     1 Arean et al. (1993~ PST              28    21    49           15.7             6.9
## 2     1 Arean et al .(1993~ Reminiscence     27    21    48           16.9             9.5
## 3     2 Ayen and Hautzinge~ CBT              11    10    21            8.5             4
## 4     2 Ayen and Hautzinge~ Supportive       20    10    30           14.6             4.5
## 5     3 Bowers et al. (199~ CBT               8     8    16            9               6.1
## 6     4 Bowman, Scogin, an~ CBT              10    10    20           12.4            11.4
## # i 8 more variables: `T-HRSD-M-Post` <dbl>, `T-HRSD-SD-Post` <dbl>,
## #   `C-BDI-M-Post` <dbl>, `C-BDI-SD-Post` <dbl>, `C-HRSD-M-Post` <dbl>,
## #   `C-HRSD-SD-Post` <dbl>, MissHRSD <chr>, MissBDI <chr>
```

We calculate the mean difference and the pooled standard deviation for each study.

```
df <- d
df$BDI_MD <- df$`T-BDI-M-Post` - df$`C-BDI-M-Post`
df$HRSD_MD <- df$`T-HRSD-M-Post` - df$`C-HRSD-M-Post`

Spooled <- function(Nt, sdt, Nc, sdc)
```

```
{
  sqrt(((Nt-1)*sdt^2 + (Nc-1)*sdc^2)/((Nt-1)+(Nc-1)))
}

BDI_sp <- Spooled(df$Nt, df$`T-BDI-SD-Post`, df$Nc, df$`C-BDI-SD-Post`)
df$BDI_SE <- BDI_sp * sqrt((1/df$Nt)+(1/df$Nc))


HRSD_sp <- Spooled(df$Nt, df$`T-HRSD-SD-Post`, df$Nc, df$`C-HRSD-SD-Post`)
df$HRSD_SE <- HRSD_sp * sqrt((1/df$Nt)+(1/df$Nc))


df_best <- df %>%
  group_by(ID) %>%
  arrange(desc(abs(HRSD_MD)), desc(abs(BDI_MD))) %>%
  slice(1) %>%
  ungroup()
```

Univariate meta-analysis

```
library(metafor)
mBDI <- rma(yi=df_best$BDI_MD, sei = df_best$BDI_SE)
```

```
## Warning: 7 studies with NAs omitted from model fitting.
```

```
summary(mBDI)
```

```
##
## Random-Effects Model (k = 30; tau^2 estimator: REML)
##
##   logLik  deviance       AIC       BIC      AICc
## -88.3246  176.6492  180.6492  183.3838  181.1107
##
## tau^2 (estimated amount of total heterogeneity): 19.4463 (SE = 6.9624)
## tau (square root of estimated tau^2 value):      4.4098
## I^2 (total heterogeneity / total variability):   77.51%
## H^2 (total variability / sampling variability):  4.45
##
## Test for Heterogeneity:
## Q(df = 29) = 160.4917, p-val < .0001
##
## Model Results:
##
## estimate      se     zval    pval    ci.lb    ci.ub
##  -7.2324  0.9469  -7.6380  <.0001  -9.0883  -5.3766  ***
##
## ---
## Signif. codes:  0 '***' 0.001 '**' 0.01 '*' 0.05 '.' 0.1 ' ' 1
```

```
mHRSD <- rma(yi=df_best$HRSD_MD, sei = df_best$HRSD_SE)
```

```
## Warning: 5 studies with NAs omitted from model fitting.
```

```
summary(mHRSD)
```

```
##
## Random-Effects Model (k = 32; tau^2 estimator: REML)
##
##   logLik  deviance       AIC       BIC      AICc
## -84.8347  169.6694  173.6694  176.5374  174.0980
##
## tau^2 (estimated amount of total heterogeneity): 8.3086 (SE = 3.0834)
## tau (square root of estimated tau^2 value):      2.8825
## I^2 (total heterogeneity / total variability):   71.92%
## H^2 (total variability / sampling variability):  3.56
##
## Test for Heterogeneity:
## Q(df = 31) = 109.7099, p-val < .0001
##
## Model Results:
##
## estimate      se      zval    pval    ci.lb    ci.ub
##  -6.3576  0.6222  -10.2183  <.0001  -7.5770  -5.1381  ***
##
## ---
## Signif. codes:  0 '***' 0.001 '**' 0.01 '*' 0.05 '.' 0.1 ' ' 1
```

Multivariate meta-analysis

```
library(mixmeta)
cor2cov <- function(sd1, sd2, cor){sd1*sd2*cor}
cor = 0.6
theta = cbind(df_best$BDI_MD, df_best$HRSD_MD)
Sigma = cbind(df_best$BDI_SE^2, cor2cov(df_best$BDI_SE, df_best$HRSD_SE, cor), df_best$HRSD_SE^2)

mvDEPR <- mixmeta(theta, Sigma, method="reml")
summary(mvDEPR)
```

```
## Call:  mixmeta(formula = theta, S = Sigma, method = "reml")
##
## Multivariate random-effects meta-analysis
## Dimension: 2
## Estimation method: REML
##
## Fixed-effects coefficients
##     Estimate  Std. Error         z  Pr(>|z|)  95%ci.lb  95%ci.ub
## y1   -6.7303      0.9093   -7.4020    0.0000   -8.5125   -4.9482  ***
## y2   -6.3437      0.6100  -10.3987    0.0000   -7.5393   -5.1480  ***
## ---
## Signif. codes:  0 '***' 0.001 '**' 0.01 '*' 0.05 '.' 0.1 ' ' 1
##
## Random-effects (co)variance components
##  Structure: General positive-definite
##     Std. Dev    Corr
## y1    4.3928      y1
## y2    2.9115  0.6447
##
## Multivariate Cochran Q-test for heterogeneity:
## Q = 279.6241 (df = 60), p-value = 0.0000
```

```
## I-square statistic = 78.5%
##
## 37 units, 2 outcomes, 62 observations, 2 fixed and 3 random-effects parameters
##    logLik        AIC        BIC
## -167.3081   344.6163   355.0880
```

Multivariate meta-analysis with SEM

```r
library(metaSEM)

mSEMDEPR <- meta(y = theta, v = Sigma)
plot(mSEMDEPR, univariate.polygon.width = 0.4,
     main = "Multivariate meta-analysis", axis.labels = c("BDI", "HRSD-17"))
```

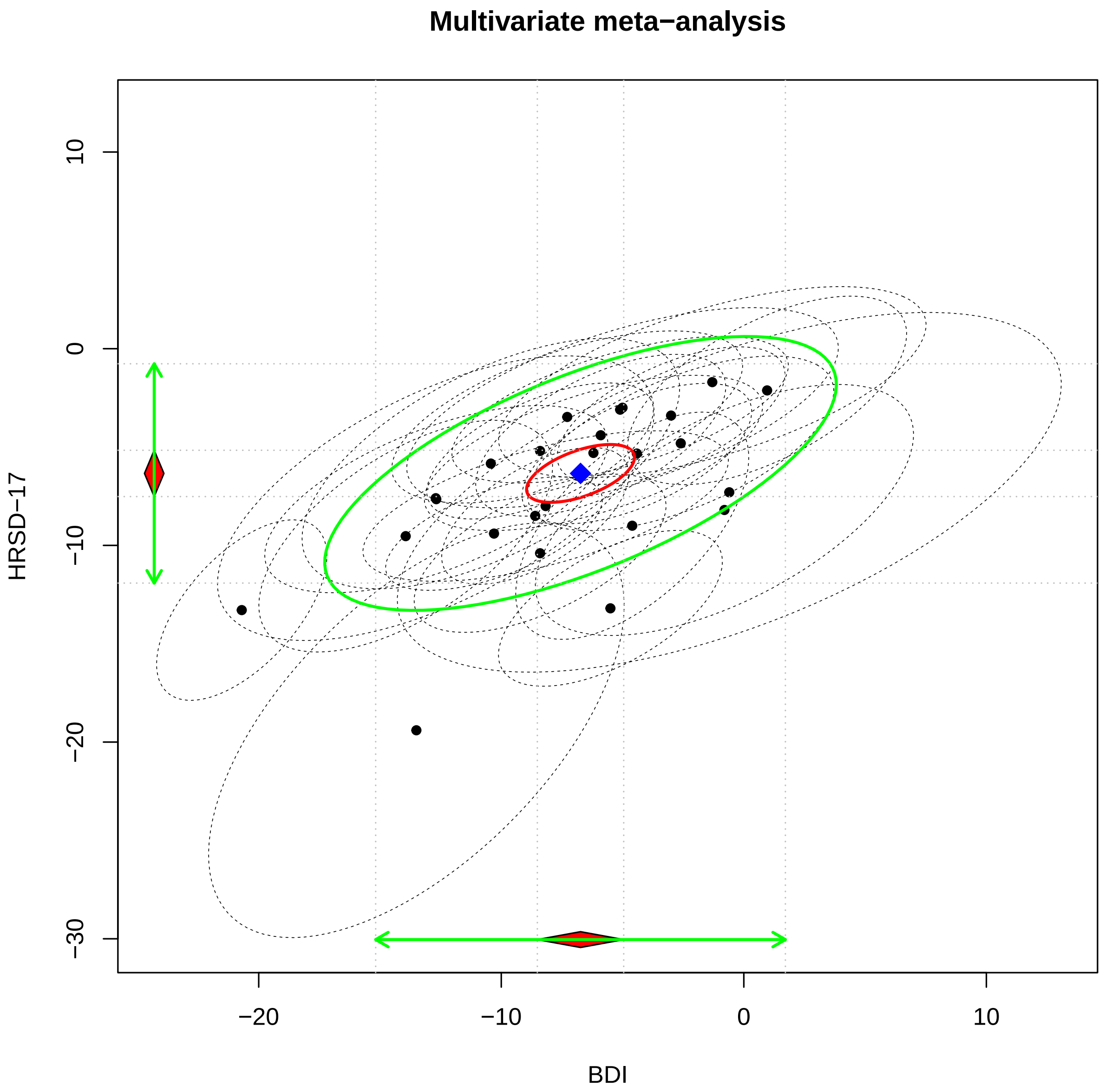

```r
mSEMDEPR <- summary(mSEMDEPR)
mSEMDEPR
```

```
##
## Call:
## meta(y = theta, v = Sigma)
##
## 95% confidence intervals: z statistic approximation (robust=FALSE)
## Coefficients:
##             Estimate Std.Error   lbound   ubound  z value  Pr(>|z|)
## Intercept1 -6.73217   0.90870 -8.51320 -4.95114  -7.4085 1.277e-13 ***
## Intercept2 -6.34113   0.60083 -7.51874 -5.16353 -10.5540 < 2.2e-16 ***
## Tau2_1_1   18.57055   6.07022  6.67315 30.46796   3.0593  0.002219 **
## Tau2_2_1    7.96742   3.64520  0.82297 15.11187   2.1857  0.028835 *
## Tau2_2_2    8.09087   3.01558  2.18045 14.00130   2.6830  0.007296 **
## ---
## Signif. codes:  0 '***' 0.001 '**' 0.01 '*' 0.05 '.' 0.1 ' ' 1
##
## Q statistic on the homogeneity of effect sizes: 279.6241
## Degrees of freedom of the Q statistic: 60
## P value of the Q statistic: 0
##
## Heterogeneity indices (based on the estimated Tau2):
##                               Estimate
## Intercept1: I2 (Q statistic)   0.7669
## Intercept2: I2 (Q statistic)   0.7138
##
## Number of studies (or clusters): 37
## Number of observed statistics: 62
## Number of estimated parameters: 5
## Degrees of freedom: 57
## -2 log likelihood: 343.583
## OpenMx status1: 0 ("0" or "1": The optimization is considered fine.
## Other values may indicate problems.)
```

Meta-analysis imputing missing outcomes

```r
library(mice)
```

```
## Warning in check_dep_version(): ABI version mismatch:
## lme4 was built with Matrix ABI version 2
## Current Matrix ABI version is 0
## Please re-install lme4 from source or restore original 'Matrix' package
```

```r
library(VIM)

dimp <- with(df_best, cbind(BDI_MD, HRSD_MD, BDI_SE, HRSD_SE))
marginplot(dimp[, c("BDI_MD", "HRSD_MD")])
```

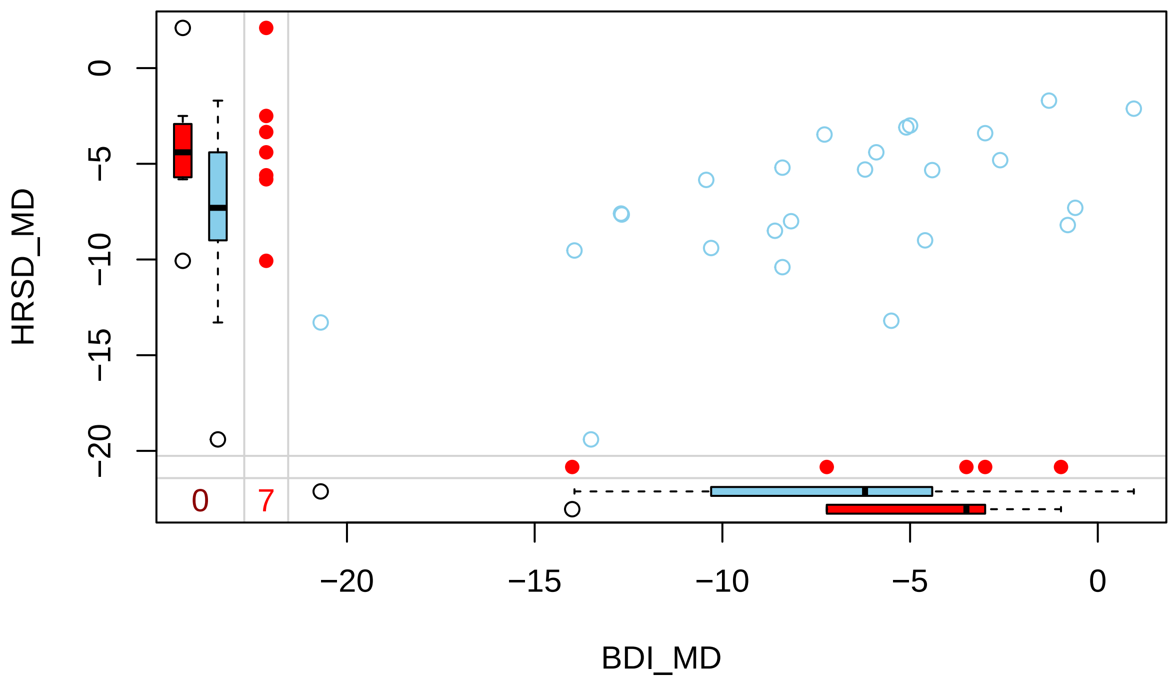


```
predMatrix <- make.predictorMatrix(dimp)
predMatrix
```

```
##          BDI_MD HRSD_MD BDI_SE HRSD_SE
## BDI_MD        0       1      1       1
## HRSD_MD       1       0      1       1
## BDI_SE        1       1      0       1
## HRSD_SE       1       1      1       0
```

```
predMatrix[, "BDI_SE"] <- 0
predMatrix[, "HRSD_SE"] <- 0
predMatrix
```

```
##          BDI_MD HRSD_MD BDI_SE HRSD_SE
## BDI_MD        0       1      0       0
## HRSD_MD       1       0      0       0
## BDI_SE        1       1      0       0
## HRSD_SE       1       1      0       0
```

```
imp <- mice(dimp, print=FALSE, m = 50, predictorMatrix = predMatrix,
            method="pmm", seed=123)

stripplot(imp, pch=20,cex=1.2)
```

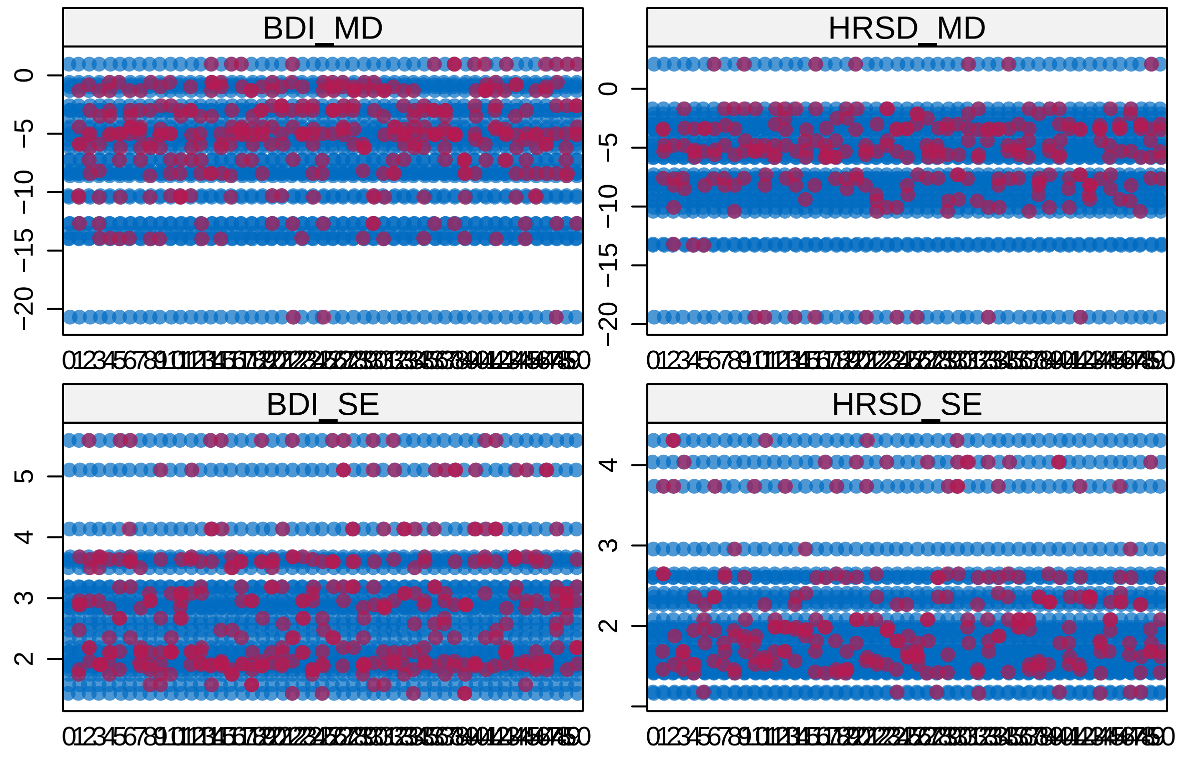


```
fit_BDI <- with(imp, rma(BDI_MD, BDI_SE^2))
pool_BDI <- summary(pool(fit_BDI))
pool_BDI
```

```
##      term  estimate std.error statistic      df      p.value
## 1 overall -6.935799  0.878761 -7.892703 30.2246 7.854002e-09
```

```
fit_HRSD <- with(imp, rma(HRSD_MD, HRSD_SE^2))
pool_HRSD <- summary(pool(fit_HRSD))
pool_HRSD
```

```
##      term  estimate std.error statistic       df      p.value
## 1 overall -6.275906 0.6115687 -10.26198 30.80675 1.848576e-11
```

Meta-analysis imputing missing outcomes with delta-adjustments

```
delta = c(-1, -0.5, 0,  0.5, 1)

adj_mnar <- list()

for (d in seq_along(delta)) {
  imp_adj <- imp

  for (i in seq_along(imp_adj$imp$BDI_MD)) {
    imp_adj$imp$BDI_MD[[i]] <- imp_adj$imp$BDI_MD[[i]] + delta[d]
    imp_adj$imp$HRSD_MD[[i]] <- imp_adj$imp$HRSD_MD[[i]] + delta[d]
  }
```

```r
  adj_mnar[[d]] <- imp_adj
}
names(adj_mnar) <- paste0("delta_", delta)
adj_mnar
```

```
## $`delta_-1`
## Class: mids
## Number of multiple imputations:  50
## Imputation methods:
##  BDI_MD HRSD_MD  BDI_SE HRSD_SE
##   "pmm"   "pmm"   "pmm"   "pmm"
## PredictorMatrix:
##         BDI_MD HRSD_MD BDI_SE HRSD_SE
## BDI_MD       0       1      0       0
## HRSD_MD      1       0      0       0
## BDI_SE       1       1      0       0
## HRSD_SE      1       1      0       0
##
## $`delta_-0.5`
## Class: mids
## Number of multiple imputations:  50
## Imputation methods:
##  BDI_MD HRSD_MD  BDI_SE HRSD_SE
##   "pmm"   "pmm"   "pmm"   "pmm"
## PredictorMatrix:
##         BDI_MD HRSD_MD BDI_SE HRSD_SE
## BDI_MD       0       1      0       0
## HRSD_MD      1       0      0       0
## BDI_SE       1       1      0       0
## HRSD_SE      1       1      0       0
##
## $delta_0
## Class: mids
## Number of multiple imputations:  50
## Imputation methods:
##  BDI_MD HRSD_MD  BDI_SE HRSD_SE
##   "pmm"   "pmm"   "pmm"   "pmm"
## PredictorMatrix:
##         BDI_MD HRSD_MD BDI_SE HRSD_SE
## BDI_MD       0       1      0       0
## HRSD_MD      1       0      0       0
## BDI_SE       1       1      0       0
## HRSD_SE      1       1      0       0
##
## $delta_0.5
## Class: mids
## Number of multiple imputations:  50
## Imputation methods:
##  BDI_MD HRSD_MD  BDI_SE HRSD_SE
##   "pmm"   "pmm"   "pmm"   "pmm"
## PredictorMatrix:
##         BDI_MD HRSD_MD BDI_SE HRSD_SE
## BDI_MD       0       1      0       0
```

```
## HRSD_MD      1       0      0       0
## BDI_SE       1       1      0       0
## HRSD_SE      1       1      0       0
##
## $delta_1
## Class: mids
## Number of multiple imputations:  50
## Imputation methods:
##  BDI_MD HRSD_MD  BDI_SE HRSD_SE
##   "pmm"   "pmm"   "pmm"   "pmm"
## PredictorMatrix:
##         BDI_MD HRSD_MD BDI_SE HRSD_SE
## BDI_MD       0       1      0       0
## HRSD_MD      1       0      0       0
## BDI_SE       1       1      0       0
## HRSD_SE      1       1      0       0
```

```r
resBDI_delta <- lapply(adj_mnar, function(met) {
  with(met, rma(BDI_MD, BDI_SE^2, method = "REML"))
})


poolBDI_delta <- lapply(resBDI_delta, pool)
sumBDI_delta <- lapply(poolBDI_delta, summary)
names(sumBDI_delta) <- names(adj_mnar)
sumBDI_delta
```

```
## $`delta_-1`
##      term  estimate std.error statistic       df      p.value
## 1 overall -7.124913 0.8729358 -8.162012 30.18715 3.936679e-09
##
## $`delta_-0.5`
##      term  estimate std.error statistic       df     p.value
## 1 overall -7.030681 0.8752908 -8.032395 30.20242 5.48567e-09
##
## $delta_0
##      term  estimate std.error statistic      df      p.value
## 1 overall -6.935799  0.878761 -7.892703 30.2246 7.854002e-09
##
## $delta_0.5
##      term  estimate std.error statistic      df      p.value
## 1 overall -6.840285 0.8833288 -7.743758 30.2539 1.153609e-08
##
## $delta_1
##      term  estimate std.error statistic       df      p.value
## 1 overall -6.744161 0.8889747  -7.58645 30.29047 1.735379e-08
```

```r
resHRSD_delta <- lapply(adj_mnar, function(met) {
  with(met, rma(HRSD_MD, HRSD_SE^2, method = "REML"))
})


poolHRSD_delta <- lapply(resHRSD_delta, pool)
sumHRSD_delta <- lapply(poolHRSD_delta, summary)
names(sumHRSD_delta) <- names(adj_mnar)
```

```r
sumHRSD_delta
```

```
## $`delta_-1`
##      term  estimate std.error statistic       df      p.value
## 1 overall -6.406902 0.6117286 -10.47344 30.73091 1.162717e-11
##
## $`delta_-0.5`
##      term  estimate std.error statistic       df      p.value
## 1 overall -6.341162 0.6108887 -10.38023 30.76628 1.423995e-11
##
## $delta_0
##      term  estimate std.error statistic       df      p.value
## 1 overall -6.275906 0.6115687 -10.26198 30.80675 1.848576e-11
##
## $delta_0.5
##      term  estimate std.error statistic       df      p.value
## 1 overall -6.211108 0.6137679 -10.11964 30.85133 2.542619e-11
##
## $delta_1
##      term  estimate std.error statistic       df      p.value
## 1 overall -6.146734 0.6174713 -9.954688 30.89923 3.699326e-11
```

```r
plot_data <- data.frame(
  Delta = rep(delta, times = 2),
  Estimate = c(
    sapply(sumBDI_delta, function(x) x$estimate),
    sapply(sumHRSD_delta, function(x) x$estimate)
  ),
  SE = c(
    sapply(sumBDI_delta, function(x) x$std.error),
    sapply(sumHRSD_delta, function(x) x$std.error)
  ),
  Outcome = rep(c("BDI", "HRSD"), each = length(delta))
) %>%
  mutate(
    Lower = Estimate - 1.96 * SE,
    Upper = Estimate + 1.96 * SE
  )

library(ggplot2)
ggsave("delta_plot.png", dpi = 300, width = 7, height = 5)
ggplot(plot_data, aes(x = Delta, y = Estimate, color = Outcome)) +
  geom_segment(aes(xend = Delta, y = Estimate - SE, yend = Estimate + SE),
               size = 0.6, alpha = 0.5) +
  geom_point(size = 3) +
  geom_line(size = 1.2) +
  facet_wrap(~ Outcome, ncol = 1, scales = "free_y") +
  scale_color_manual(values = c("BDI" = "#647AA3", "HRSD" = "#80475E")) +
  theme_minimal(base_size = 13) +
  labs(
    title = "Delta-adjusted pooled estimates",
    x = expression(paste("Delta adjustment (", delta, ")")),
    y = "Pooled Estimate"
  ) +
```

```r
  theme(
    strip.text = element_text(face = "bold"),
    panel.grid.minor = element_blank(),
    legend.position = "none"
  )
```

```
## Warning: Using `size` aesthetic for lines was deprecated in ggplot2 3.4.0.
## i Please use `linewidth` instead.
## This warning is displayed once every 8 hours.
## Call `lifecycle::last_lifecycle_warnings()` to see where this warning was generated.
```

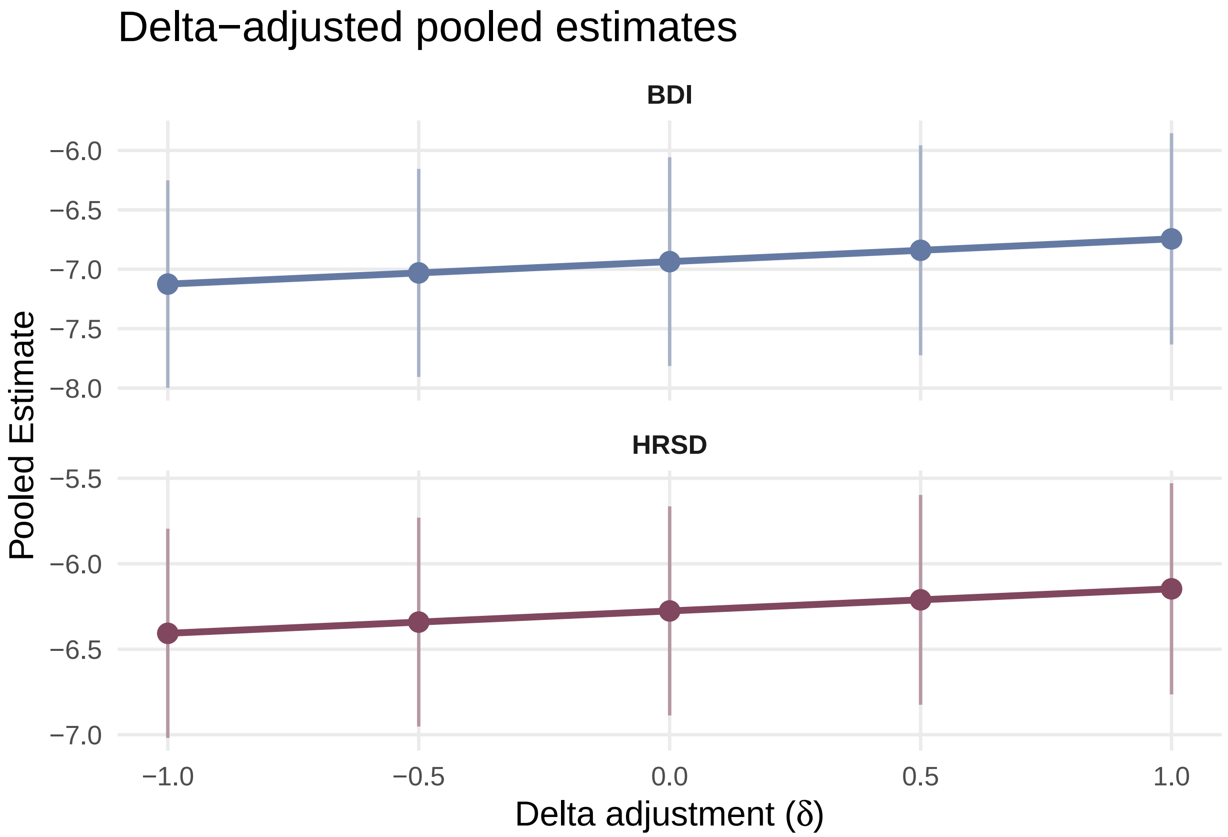


```r
dev.off()
```

```
## null device
##           1
```
