# Supplementary material for: Change of d-irection: current limitations and future directions in psychological meta-analysis
Source: Front Psychol. 2026 Feb 13;17:1717798. doi: 10.3389/fpsyg.2026.1717798 (PMC12946090; doi:10.3389/fpsyg.2026.1717798)
Supplement: Supplementary file 5 [file Data_Sheet_5.PDF]

R code for simulation study - Multivariate with wrong correlation

2025-12-21

```
rm(list = ls())

library(metafor)
library(mvtnorm)
library(systemfit)
library(dplyr)
library(parallel)
library(furrr)
library(purrr)
library(mixmeta)
library(mice)

# With this function we simulate the individual participant study data, we
# estimate the treatment effects for each study, generate missing data according
# to MCAR MAR and MNAR mechanisms and perform the meta-analysis.
# Specific comments are inserted directly in the function.

# We did not set any random seed.

sim_miss <- function(iter,
                     target = 0.30,
                     S = 50,
                     true_eff = c(3, 5),
                     mechanism = c("None", "MCAR", "MAR", "MNAR"),
                     imprho = NA_real_) # set rho below
  {
  mechanism <- match.arg(mechanism)

  # Draw random treatment effects per study (for the random-effects meta-analysis)
  Mu.CR <- c(true_eff[1], 0)
  Mu.SR <- c(true_eff[2], 0)
  Tau <- c(1, 1)
  rho <- 0
  Sigma <- diag(Tau) %*% matrix(c(1, rho, rho, 1), 2) %*% diag(Tau)

  Eff.CR <- mvtnorm::rmvnorm(S, Mu.CR, Sigma)
  Eff.SR <- mvtnorm::rmvnorm(S, Mu.SR, Sigma)

  New.CR <- Eff.CR[, 1]
  Std.CR <- Eff.CR[, 2]

  New.SR <- Eff.SR[, 1]
  Std.SR <- Eff.SR[, 2]
```

```
Noise <- c(CR = 2, SR = 8)

# Generate the single studies for the meta analysis
data <- lapply(seq_len(S), function(i) {
  N <- sample(40:100, 1) # these are the participants in each study
  Age <- runif(N, min = 20, max = 80)
  Sex <- factor(rbinom(N, 1, 0.45),
                levels = 0:1,
                labels = c("M", "F"))
  Therapy <- factor(sample(c("New", "Std"), N, replace = TRUE))

  # the latent illness, for example depression
  Ill <- rnorm(N, 20 + 1 * as.numeric(Sex) + 0.5 * Age, 5)
  Mood.T0 <- rnorm(N, 0, 5)

  T0.CR <- Ill + Mood.T0 + rnorm(N, 0, Noise["CR"])
  T0.SR <- Ill + Mood.T0 + rnorm(N, 0, Noise["SR"])

  Effs.CR <- c(New.CR = New.CR[i], Std.CR = Std.CR[i])
  Effs.SR <- c(New.SR = New.SR[i], Std.SR = Std.SR[i])

  Latent.CR <- Ill + Effs.CR[Therapy]
  Latent.SR <- Ill + Effs.SR[Therapy]
  Mood.EOT <- rnorm(N, 0, 5)

  # final outcome measure of the latent illness on the CR (clinician rating)
  # and SR (self report)
  CR <- Ill + Effs.CR[Therapy] + Mood.EOT + rnorm(N, 0, Noise["CR"])
  SR <- Ill + Effs.SR[Therapy] + Mood.EOT + rnorm(N, 0, Noise["SR"])

  data.frame(
    Study = i,
    Age = Age,
    Sex = Sex,
    Therapy = Therapy,
    T0.CR = T0.CR,
    T0.SR = T0.SR,
    CR = CR,
    SR = SR
  )
})

d <- bind_rows(data)

# Fit SUR to obtain summary estimates for meta-analysis (we use systemfit)
dat <- bind_rows(lapply(seq_len(S), function(s) {
  Sn <- filter(d, Study == s)
  Sn$Therapy <- relevel(Sn$Therapy, ref = "Std")
  fit <- systemfit(list(
    CR = CR ~ Age + Sex + T0.CR + Therapy,
    SR = SR ~ Age + Sex + T0.SR + Therapy
  ),
  "SUR",
```

```r
    data = Sn)
  sum <- summary(fit)

  tibble(
    Study = s,
    EstCR = sum$coefficients[5, 1],
    SECR = sum$coefficients[5, 2],
    EstSR = sum$coefficients[10, 1],
    SESR = sum$coefficients[10, 2],
    Cor.ws = sum$residCor["CR", "SR"]
  )
}))

# Apply missing data mechanism

if (mechanism == "None") {
  dmiss <- dat  # no missingness, complete data
}

# Generate Missing Completely At Random

else if (mechanism == "MCAR") {
  size <- round(S * target / 2)
  M_CR <- sample(S, size, replace = FALSE)
  M_SR <- sample(setdiff(seq_len(S), M_CR), size, replace = FALSE)
  dmiss <- dat %>%
    mutate(
      EstCR = if_else(Study %in% M_CR, NA, EstCR),
      SECR  = if_else(Study %in% M_CR, NA, SECR),
      EstSR = if_else(Study %in% M_SR, NA, EstSR),
      SESR  = if_else(Study %in% M_SR, NA, SESR),
      Cor.ws = if_else(Study %in% union(M_CR, M_SR), NA, Cor.ws)
    )
}

# Generate Missing At Random (only for CR, dependent on sample size N)

else if (mechanism == "MAR") {
  sub <- d %>%
    group_by(Study) %>%
    summarise(N = n(), .groups = "drop")

  invlogit <- plogis

  Nc <- scale(sub$N, center = TRUE, scale = FALSE)[, 1]

  betaN_cr <- 0.25

  beta0_cr <- uniroot(function(b0)
    mean(invlogit(b0 + betaN_cr * Nc)) - (1 - target / 2),
    interval = c(-20, 20))$root

  sub <- sub %>%
```

```r
    mutate(
      p_obs_CR = invlogit(beta0_cr + betaN_cr * Nc),
      p_obs_SR = 1 - target / 2,
      M_CR = rbinom(n(), 1, p_obs_CR),
      M_SR = rbinom(n(), 1, p_obs_SR)
    )

  conflict <- which(sub$M_CR == 0 & sub$M_SR == 0)
  if (length(conflict) > 0) {
    meanCR_obs <- mean(dat$EstCR, na.rm = TRUE)
    meanSR_obs <- mean(dat$EstSR, na.rm = TRUE)
    for (i in conflict) {
      d_i <- dat[i, ]
      dist_cr <- abs(d_i$EstCR - meanCR_obs)
      dist_sr <- abs(d_i$EstSR - meanSR_obs)
      if (dist_cr > dist_sr) {
        sub$M_CR[i] <- 1
      } else {
        sub$M_SR[i] <- 1
      }
    }
  }

  dmiss <- dat %>%
    left_join(sub %>% select(Study, M_CR, M_SR), by = "Study") %>%
    mutate(
      EstCR = if_else(M_CR == 0, NA, EstCR),
      SECR  = if_else(M_CR == 0, NA, SECR),
      EstSR = if_else(M_SR == 0, NA, EstSR),
      SESR  = if_else(M_SR == 0, NA, SESR),
      Cor.ws = if_else(is.na(EstCR) | is.na(EstSR), NA, Cor.ws)
    )
}

# Generate Missing Not At Random (0.62 was manually determined to create the
# desired target of 40% missing in each outcome)

else if (mechanism == "MNAR") {
  invlogit <- plogis
  betaCR <- 2
  betaSR <- 1

  if (target == 0.80) {
    mnar_const <- 0.62
  } else if (target == 0.40) {
    mnar_const <- 0.38
  }

  beta0_cr <- uniroot(function(b0)
    mean(invlogit(b0 + betaCR * dat$EstCR)) - (1 - mnar_const),
    interval = c(-20, 20))$root
  prob_cr <- invlogit(beta0_cr + betaCR * dat$EstCR)
```

```
  beta0_sr <- uniroot(function(b0)
    mean(invlogit(b0 + betaSR * dat$EstSR)) - (1 - mnar_const),
    interval = c(-20, 20))$root
  prob_sr <- invlogit(beta0_sr + betaSR * dat$EstSR)

  M_cr <- rbinom(nrow(dat), 1, 1 - prob_cr)
  M_sr <- rbinom(nrow(dat), 1, 1 - prob_sr)

  conflict <- which(M_cr == 1 & M_sr == 1)
  meanCR_obs <- mean(dat$EstCR, na.rm = TRUE)
  meanSR_obs <- mean(dat$EstSR, na.rm = TRUE)

  # more severe for the self-report in case of conflicts

  for (i in conflict) {
    dist_cr <- abs(dat$EstCR[i] - meanCR_obs)
    dist_sr <- abs(dat$EstSR[i] - meanSR_obs)
    if (dist_cr > dist_sr) {
      M_sr[i] <- 0
    } else {
      M_cr[i] <- 0
    }
  }

  dmiss <- dat %>%
    mutate(
      M_CR = M_cr,
      M_SR = M_sr,
      EstCR = if_else(M_CR == 1, NA, EstCR),
      SECR  = if_else(M_CR == 1, NA, SECR),
      EstSR = if_else(M_SR == 1, NA, EstSR),
      SESR  = if_else(M_SR == 1, NA, SESR),
      Cor.ws = if_else(is.na(EstCR) | is.na(EstSR), NA, Cor.ws)
    )
}

# Multivariate meta-analysis (we use mixmeta)

cor2cov <- function(sd1, sd2, rho)
  sd1 * sd2 * rho

rho_used <- if (is.na(imprho)) dmiss$Cor.ws else imprho

Sigma <- cbind(dmiss$SECR^2,
               cor2cov(dmiss$SECR, dmiss$SESR, rho_used),
               dmiss$SESR^2)
theta <- cbind(dmiss$EstCR, dmiss$EstSR)

mvfit <- mixmeta(theta, Sigma, method = "ml")
est <- coef(mvfit)
se <- sqrt(diag(vcov(mvfit)))

tibble(
```

```r
    rep = iter,
    mechanism = mechanism,
    target = target,
    imprho = imprho,
    CR_Est = est[1],
    CR_SE = se[1],
    SR_Est = est[2],
    SR_SE = se[2],
    CR_Bias = est[1] - true_eff[1],
    SR_Bias = est[2] - true_eff[2],
    CR_Coverage = between(true_eff[1], est[1] - 1.96 * se[1], est[1] + 1.96 * se[1]),
    SR_Coverage = between(true_eff[2], est[2] - 1.96 * se[2], est[2] + 1.96 * se[2])
  )
}

grid <- expand.grid(
  mechanism = c("MAR"),
  target = c(0, 0.80),
  imprho = c(NA, 0.2), # insert here the correlations
  stringsAsFactors = FALSE
)

grid <- subset(grid,
               (mechanism == "None" & target == 0) |
                 (mechanism != "None" & target > 0))
# Set the number of iterations
iter <- 1000

# Create the grid for all possible conditions of the simulation
grid_final <- grid[rep(1:nrow(grid), each = 1), ]
grid_final$nsim <- iter

safe_sim_miss <- purrr::safely(sim_miss, otherwise = NULL)

rep_sim <- function(nsim = 1, ...) {
  args <- list(...)
  lapply(seq_len(nsim), function(i) {
    message(
      sprintf(
        "Running iteration %d (mechanism = %s, target = %.2f, imprho =%s)",
        i,
        args$mechanism,
        args$target,
        ifelse(is.na(args$imprho), "SUR", as.character(args$imprho))
      )
    )
    res <- do.call(safe_sim_miss, c(args, list(iter = i)))
    if (!is.null(res$error)) {
      message(sprintf("Error in iteration %d: %s", i, res$error$message))
    }
    res
  })
}
```

```r
# We conducted the simulation on a server with 10 cores.
n_cores <- min(availableCores(), 10)
message("Using ", n_cores, " cores")
plan(multisession, workers = n_cores)

res <- future_pmap(
  grid_final,
  ~ rep_sim(
    nsim = ..4,
    imprho = ..3,
    mechanism = ..1,
    target = ..2
  ),
  .options = furrr_options(seed = TRUE),
  .progress = TRUE
)

message("Parallel processing complete")
message("Extracting and binding results...")

res_split <- lapply(res, function(x) {
  tibble::tibble(result = purrr::map(x, "result"),
                 error  = purrr::map(x, "error"))
})

res_clean <- lapply(res_split, function(df) {
  df_clean <- df %>% filter(map_lgl(error, is.null))
  map_dfr(df_clean$result, identity)
})

# We did this because we want to check if there were any errors, but not stop the
# simulation in between.

res_errors <- lapply(res_split, function(df) {
  df %>%
    filter(!map_lgl(error, is.null)) %>%
    mutate(error_msg = map_chr(error, ~ .x$message))
})

grid_final$res <- res_clean
grid_final$errors <- res_errors
```
