# Supplementary material for: Change of d-irection: current limitations and future directions in psychological meta-analysis
Source: Front Psychol. 2026 Feb 13;17:1717798. doi: 10.3389/fpsyg.2026.1717798 (PMC12946090; doi:10.3389/fpsyg.2026.1717798)
Supplement: Supplementary file 6 [file Data_Sheet_6.PDF]

Additional results from the simulation study

We present here the results from the simulation study conducted with other combinations of parameters. If the reader is interested in other settings, it is possible to download the scripts and change the parameters accordingly.

We further present how the estimate and standard errors change for multivariate meta-analysis under MAR when within-study correlations are misspecified.

50 Studies and 20% missingness at outcome level

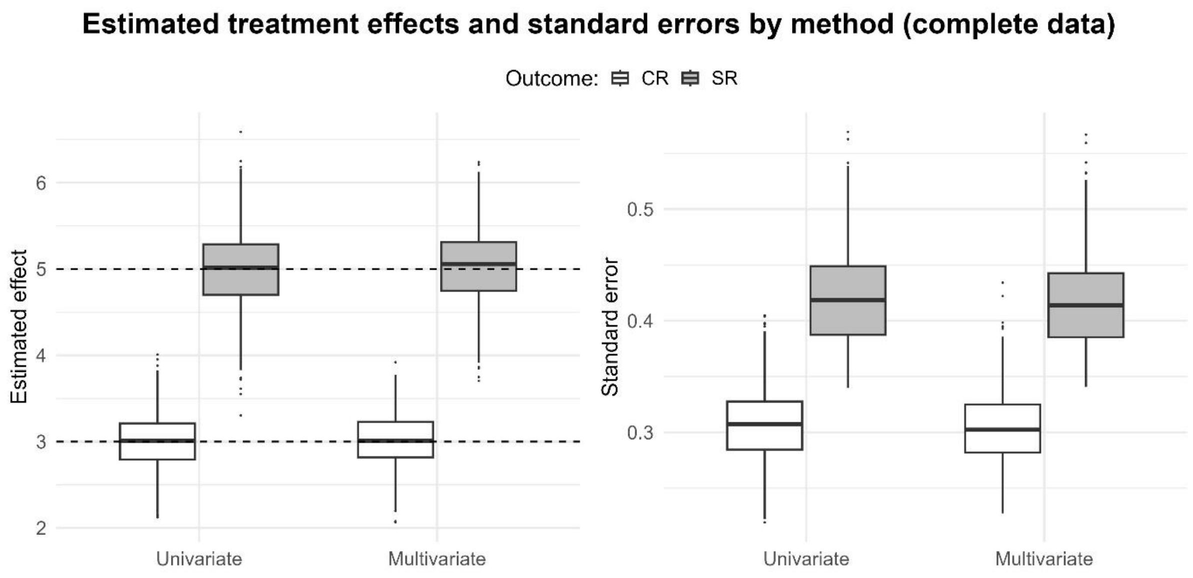

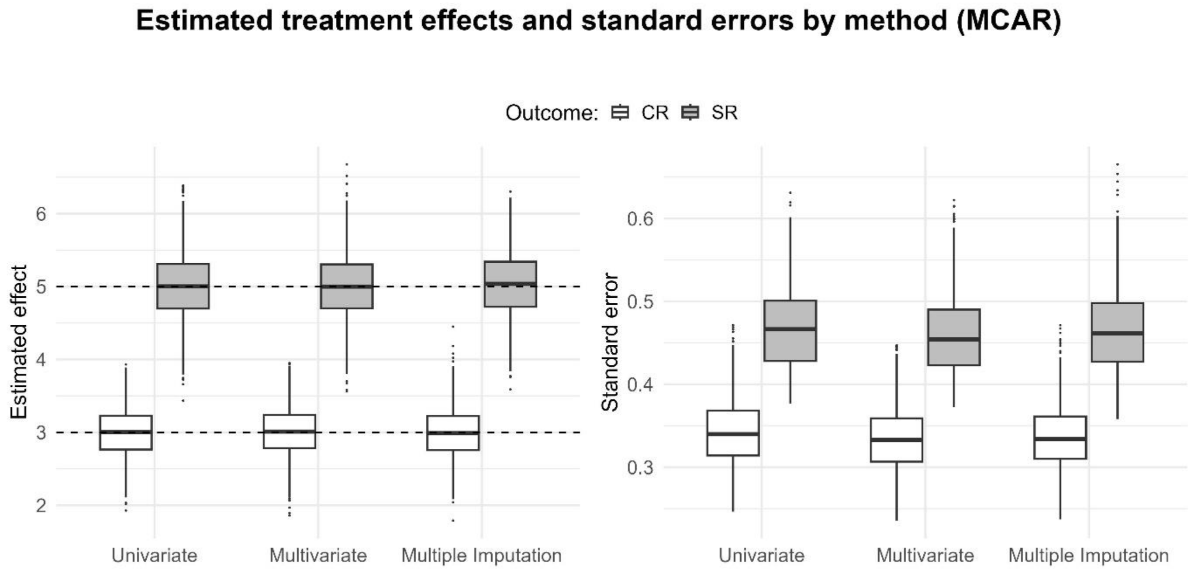

Estimated treatment effects and standard errors by method (MAR)

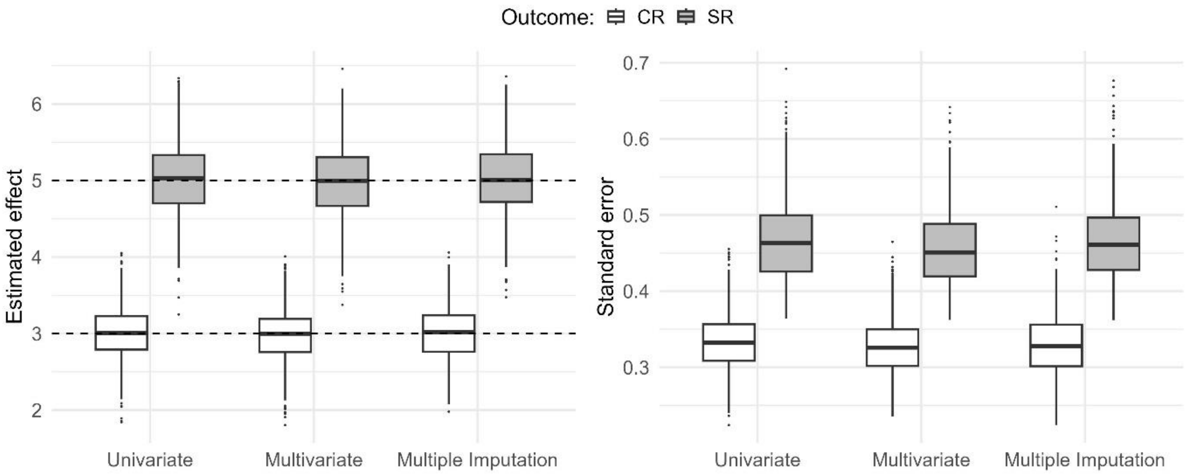

Estimated treatment effects and standard errors by method (MNAR)

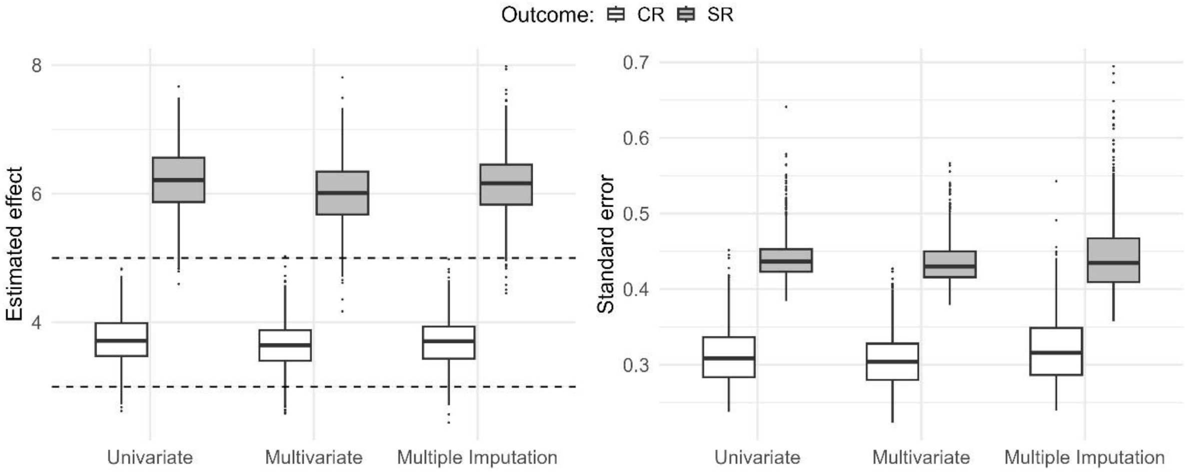

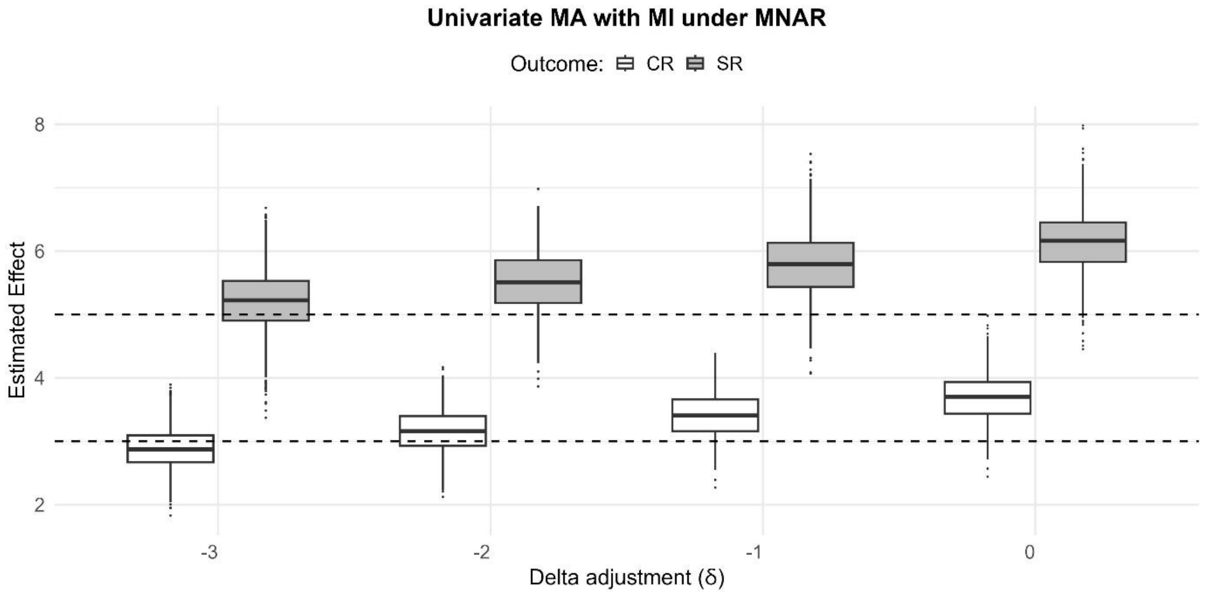

Method	Mechanism	CR Coverage	SR Coverage	CR Bias	SR Bias
Univariate	Complete	0.954	0.944	0.002	0.003
Univariate	MCAR	0.948	0.947	-0.001	0.004
Univariate	MAR	0.942	0.942	0.011	0.020
Univariate	MNAR	0.380	0.245	0.724	1.210
Multivariate	Complete	0.941	0.944	0.019	0.037
Multivariate	MCAR	0.940	0.941	0.009	0.010
Multivariate	MAR	0.938	0.942	-0.019	-0.014
Multivariate	MNAR	0.449	0.373	0.647	1.008
Multiple Imputation	MCAR	0.946	0.948	-0.010	0.028
Multiple Imputation	MAR	0.945	0.969	0.001	0.019
Multiple Imputation	MNAR	0.423	0.261	0.689	1.153

Note. Method indicates the meta-analytical strategy used; Mechanism indicates the missing data mechanism used to generate the missing values; Coverage (respectively for the clinician rating, CR and self-report SR) is defined as the proportion of replicates in which the 95% confidence interval contained the true effect; Bias is calculated as the average difference between the estimated and the true effects.

25 Studies and 40% missingness at outcome level

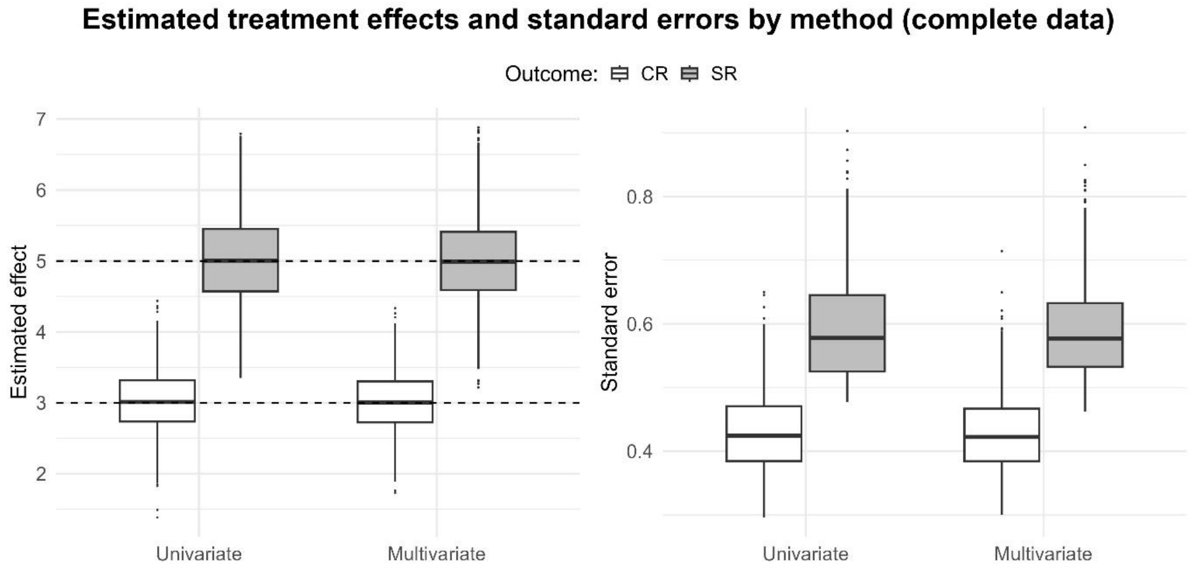

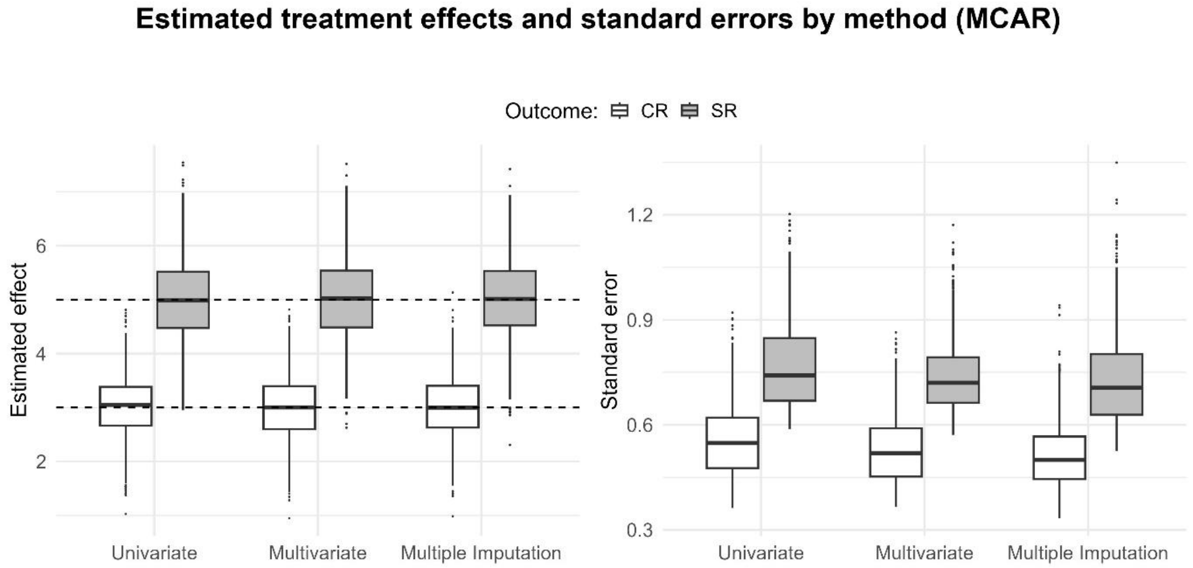

Estimated treatment effects and standard errors by method (MAR)

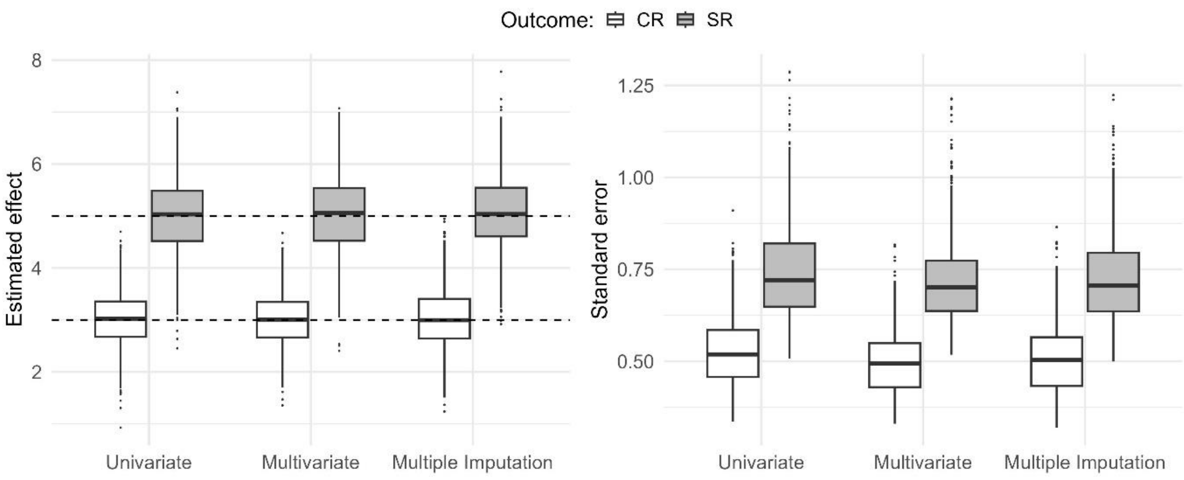

Estimated treatment effects and standard errors by method (MNAR)

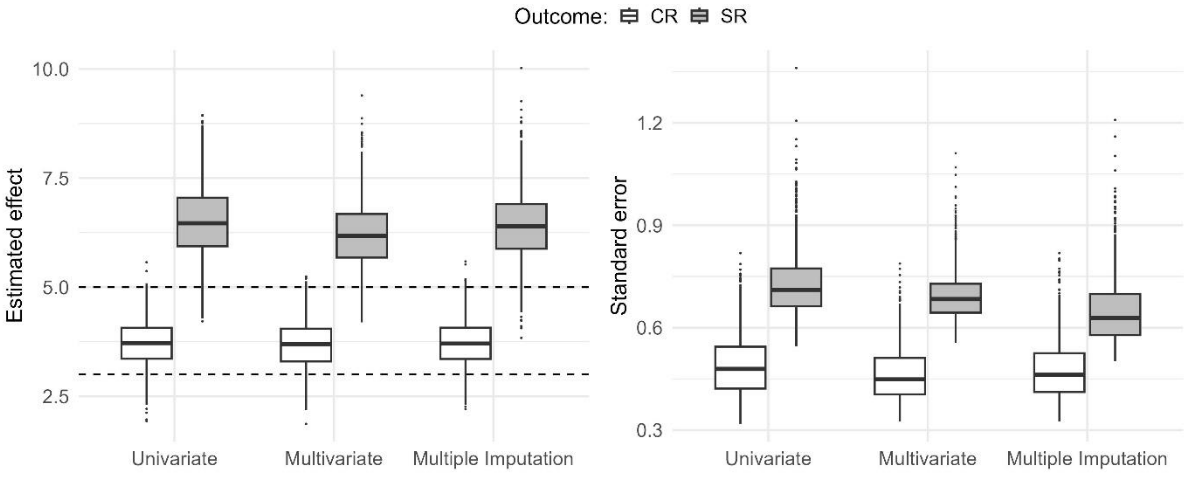

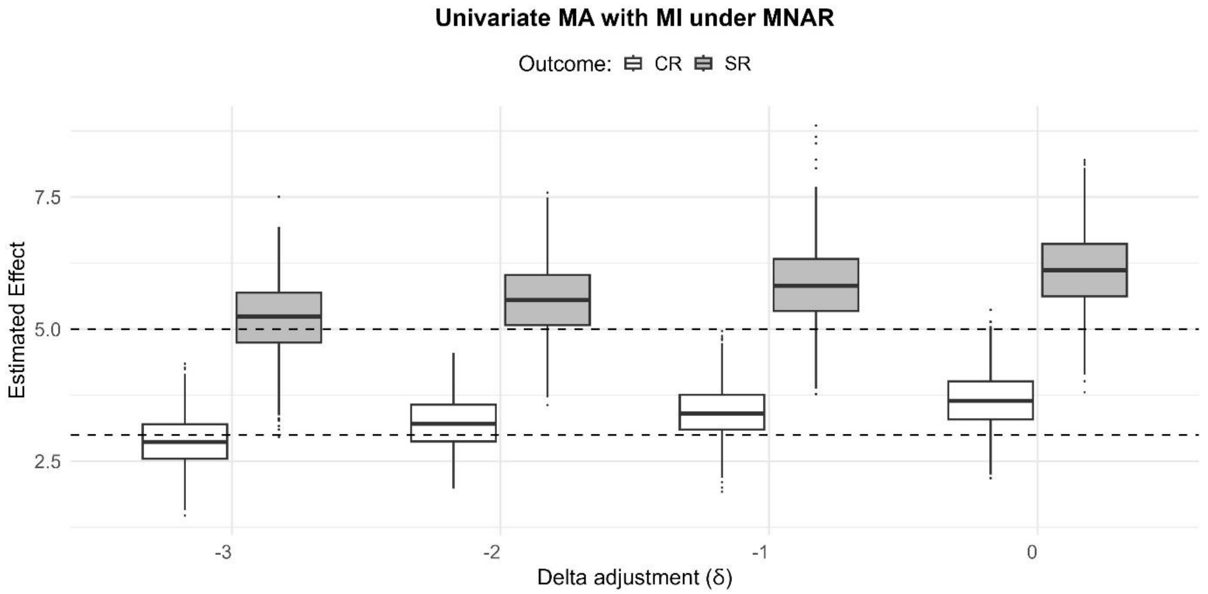

Method	Mechanism	CR Coverage	SR Coverage	CR Bias	SR Bias
Univariate	Complete	0.937	0.943	0.010	0.007
Univariate	MCAR	0.934	0.944	0.028	0.000
Univariate	MAR	0.949	0.953	0.014	0.000
Univariate	MNAR	0.679	0.463	0.723	1.498
Multivariate	Complete	0.929	0.930	0.006	0.011
Multivariate	MCAR	0.924	0.938	0.000	0.012
Multivariate	MAR	0.920	0.950	0.001	0.030
Multivariate	MNAR	0.660	0.607	0.694	1.205
Multiple Imputation	MCAR	0.910	0.926	0.013	0.023
Multiple Imputation	MAR	0.909	0.939	0.017	0.052
Multiple Imputation	MNAR	0.657	0.433	0.716	1.418

Note. Method indicates the meta-analytical strategy used; Mechanism indicates the missing data mechanism used to generate the missing values; Coverage (respectively for the clinician rating, CR and self-report SR) is defined as the proportion of replicates in which the 95% confidence interval contained the true effect; Bias is calculated as the average difference between the estimated and the true effects.

25 Studies and 20% missingness at outcome level

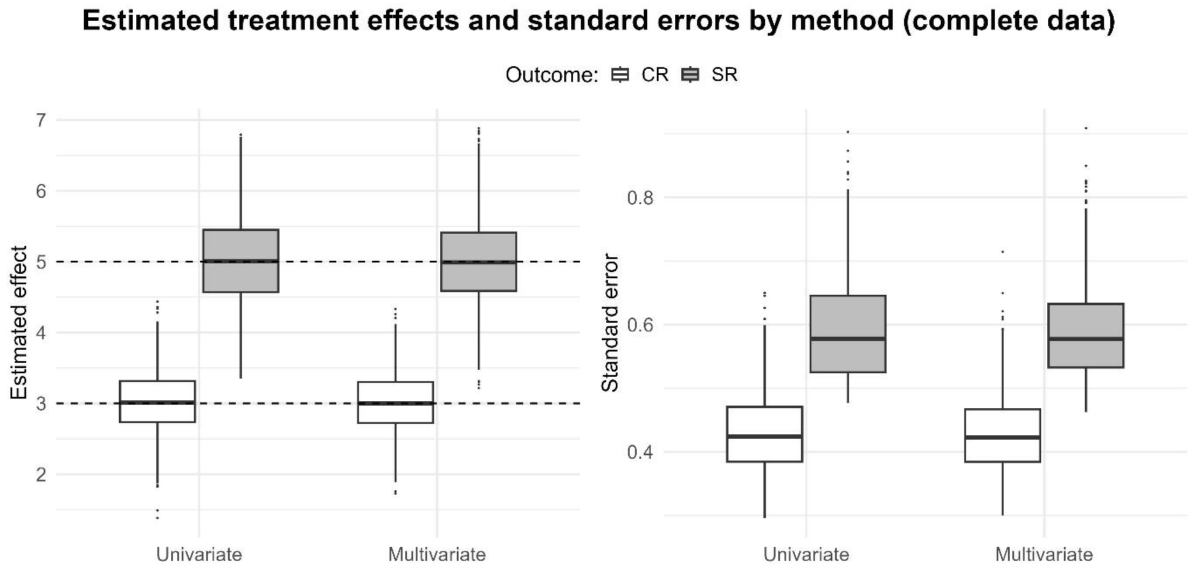

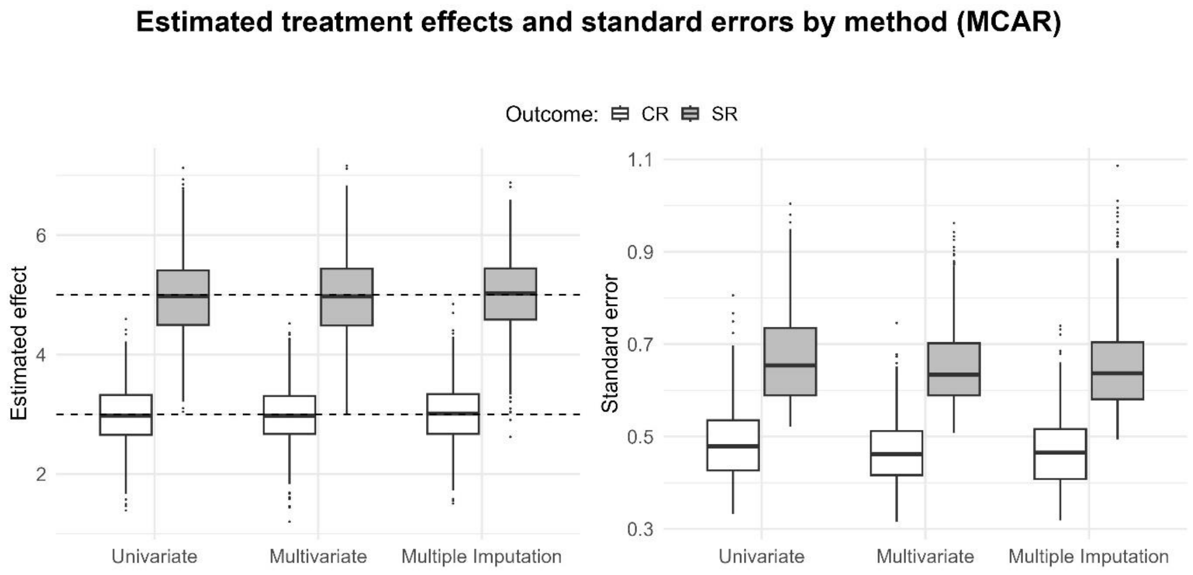

Estimated treatment effects and standard errors by method (MAR)

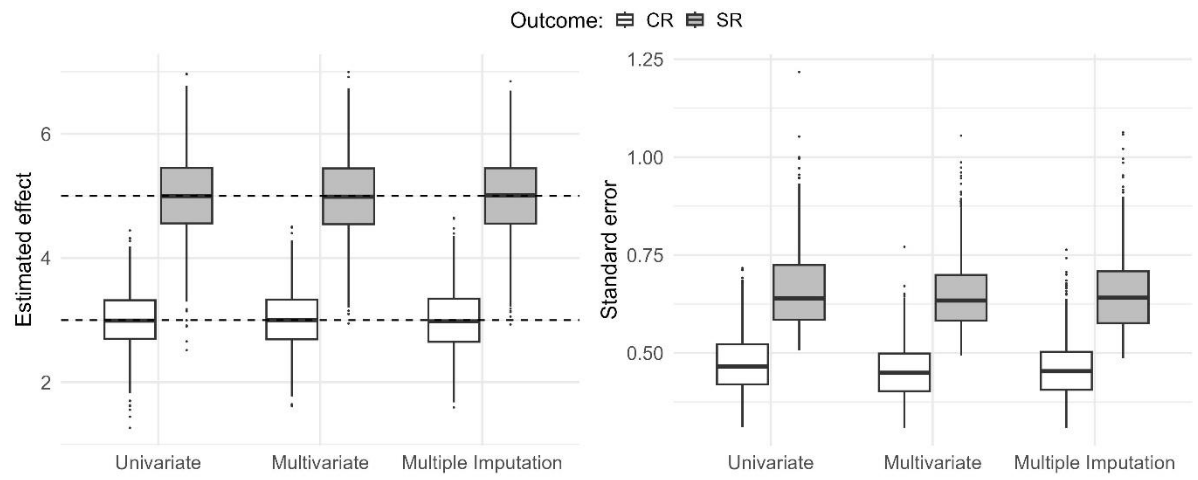

Univariate MA with MI under MNAR

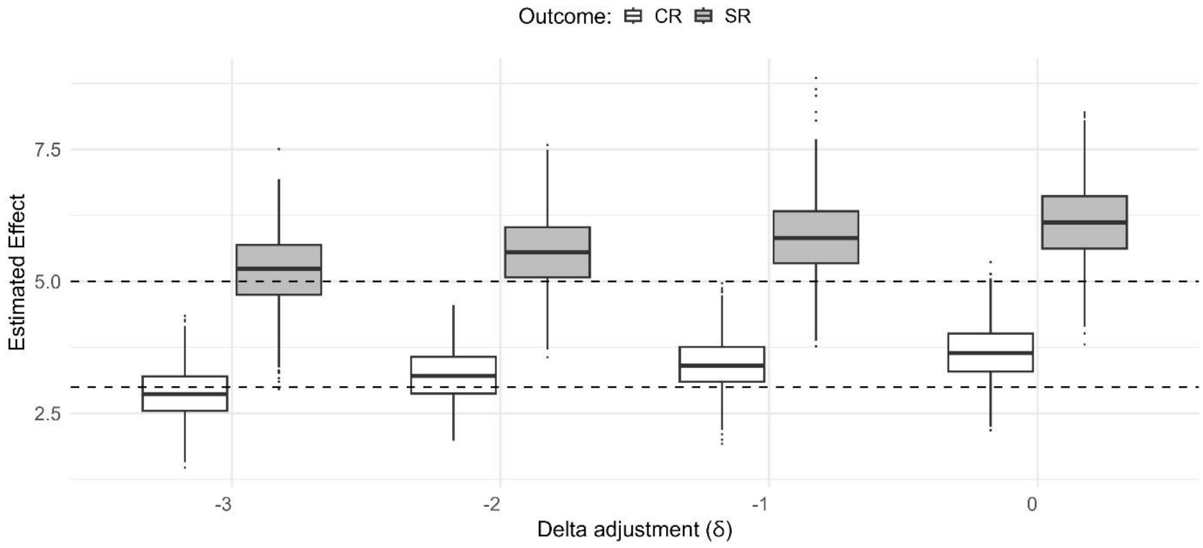

Method	Mechanism	CR Coverage	SR Coverage	CR Bias	SR Bias
Univariate	Complete	0.937	0.943	0.010	0.007
Univariate	MCAR	0.937	0.940	-0.014	-0.031
Univariate	MAR	0.937	0.935	0.003	-0.015
Univariate	MNAR	0.595	0.516	0.733	1.227
Multivariate	Complete	0.929	0.930	0.006	0.011
Multivariate	MCAR	0.924	0.929	-0.021	-0.023
Multivariate	MAR	0.943	0.951	0.004	-0.011
Multivariate	MNAR	0.663	0.626	0.658	1.021
Multiple Imputation	MCAR	0.912	0.935	0.001	-0.001
Multiple Imputation	MAR	0.922	0.939	-0.012	-0.005
Multiple Imputation	MNAR	0.640	0.531	0.656	1.108

Note. Method indicates the meta-analytical strategy used; Mechanism indicates the missing data mechanism used to generate the missing values; Coverage (respectively for the clinician rating, CR and self-report SR) is defined as the proportion of replicates in which the 95% confidence interval contained the true effect; Bias is calculated as the average difference between the estimated and the true effects.

50 Studies 40% missingness at outcome level (MAR) with misspecified correlation for multivariate meta-analysis

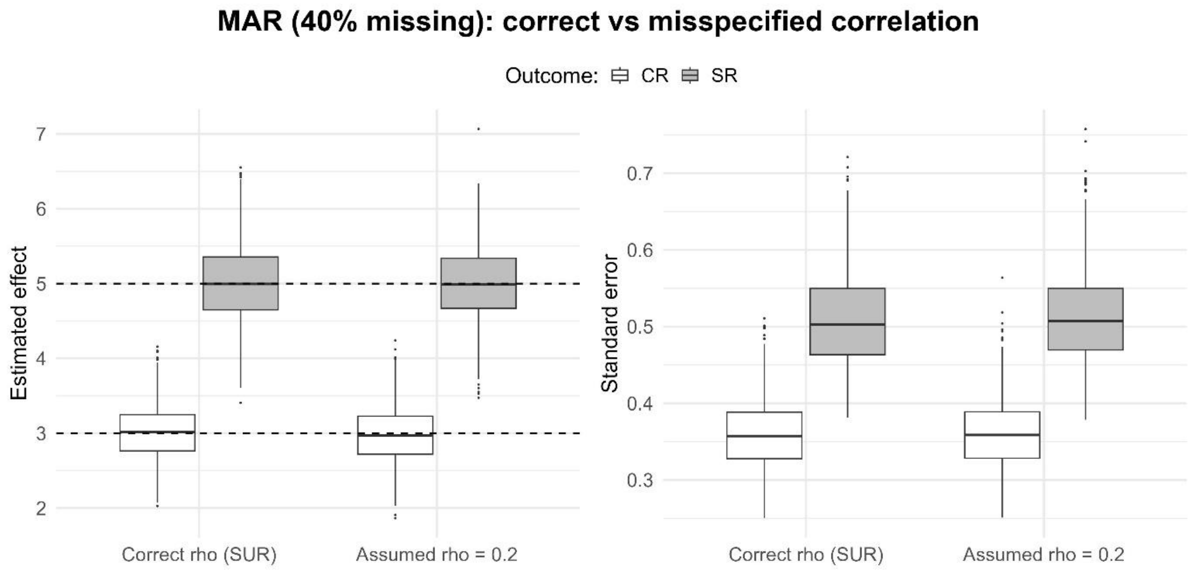

As the reader can easily notice, the estimates and the standard errors change marginally. This is consistent with what has been reported in previous studies (e.g., Ishak et al., 2008; Riley et al., 2017). Conceptually, even if some studies do not provide joint measurement, the multivariate model can still borrow information from studies with complete overlap, so the impact of misspecification can be moderate in practice.

References

Ishak, K. J., Platt, R. W., Joseph, L., and Hanley, J. A. (2008). Impact of approximating or ignoring within-study covariances in multivariate meta-analyses. *Stat Med* 27, 670–686. doi: 10.1002/sim.2913

Riley, R. D., Jackson, D., Salanti, G., Burke, D. L., Price, M., Kirkham, J., et al. (2017). Multivariate and network meta-analysis of multiple outcomes and multiple treatments: rationale, concepts, and examples. *BMJ*, j3932. doi: 10.1136/bmj.j3932
